# Quantitative Proteomic Analysis Reveals Functional Alterations of the Peripheral Immune System in Colorectal Cancer

**DOI:** 10.1016/j.mcpro.2024.100784

**Published:** 2024-05-11

**Authors:** Wenyuan Zhu, Minzhe Li, Qingsong Wang, Jian Shen, Jianguo Ji

**Affiliations:** 1 State Key Laboratory of Protein and Plant Gene Research, School of Life Sciences, Peking University, Beijing, China; 2 Department of Biochemistry and Molecular Biology, School of Life Sciences, Peking University, Beijing, China; 3 General Surgery Department, Beijing Chao-Yang Hospital, Capital Medical University, Beijing, China

**Keywords:** colorectal cancer, quantitative proteomics, peripheral immune system, ligand-receptor interaction, biomarker

## Abstract

Colorectal cancer (CRC) is characterized by high morbidity, high mortality, and limited response to immunotherapies. The peripheral immune system is an important component of tumor immunity, and enhancements of peripheral immunity help to suppress tumor progression. However, the functional alterations of the peripheral immune system in CRC are unclear. Here, we used mass spectrometry-based quantitative proteomics to establish a protein expression atlas for the peripheral immune system in CRC, including plasma and five types of immune cells (CD4^+^ T cells, CD8^+^ T cells, monocytes, natural killer cells, and B cells). Synthesizing the results of the multidimensional analysis, we observed an enhanced inflammatory phenotype in CRC, including elevated expression of plasma inflammatory proteins, activation of the inflammatory pathway in monocytes, and increased inflammation-related ligand-receptor interactions. Notably, we observed tumor effects on peripheral T cells, including altered cell subpopulation ratios and suppression of cell function. Suppression of CD4^+^ T cell function is mainly mediated by high expression levels of protein tyrosine phosphatases. Among them, the expression of protein tyrosine phosphatase receptor type J (PTPRJ) gradually increased with CRC progression; knockdown of PTPRJ *in vitro* could promote T cell activation, thereby enhancing peripheral immunity. We also found that the combination of leucine-rich α-2 glycoprotein 1 (LRG1) and apolipoprotein A4 (APOA4) had the best predictive ability for colorectal cancer and has the potential to be a biomarker. Overall, this study provides a comprehensive understanding of the peripheral immune system in CRC. It also offers insights regarding the potential clinical utilities of these peripheral immune characteristics as diagnostic indicators and therapeutic targets.

## Highlights

• Quantitative proteomic atlas of CRC peripheral blood.
• Comprehensive analysis of functional alterations in the peripheral immune system.
• In-depth understanding of immune profiles as biomarkers and therapeutic targets.

## Graphical Abstract

**Figure:**
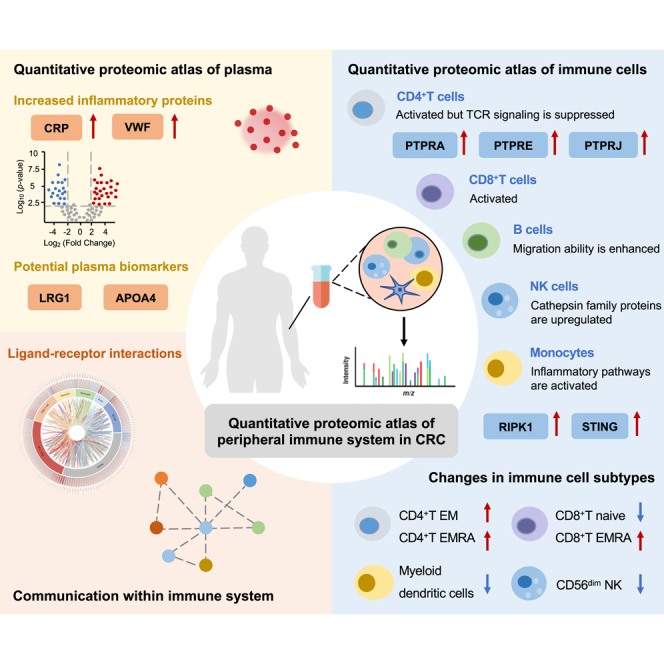

## In Brief

This study generated a proteomic atlas for the peripheral immune system in colorectal cancer (CRC), including plasma and five types of immune cells. Through multifaceted analyses including protein profiling, cellular subpopulation distribution, and receptor-ligand interactions, we identified key features of tumor remodeling of the peripheral immune system, mainly in the form of enhanced inflammation and changes in CD4^+^T cell function. These results offer insights regarding the potential clinical utilities of peripheral immune characteristics as diagnostic indicators and therapeutic targets.

Colorectal cancer (CRC) is a common malignant tumor. In recent years, its mortality rate has been high, and its incidence has increased (1). Efforts to diagnose and treat CRC are ongoing, and treatment strategies are shifting from primarily focusing on tumor cells to incorporating the broader concept of tumor immunity (2). Although immunotherapies involving immune checkpoint inhibitors have demonstrated therapeutic potential, they have not been effective in proficient mismatch repair/microsatellite instability-low (pMMR/MSI-L) patients (the largest group of patients with CRC) (3). This lack of success has arisen because existing immunotherapies depend on the expression of immune checkpoint proteins and high levels of tumor-killing T-cell infiltration. Therefore, tumor immunity must be activated to improve immunotherapy efficacy in CRC.

In recent years, the concept of an immune macroenvironment has been proposed. In addition to the tumor microenvironment, the immune macroenvironment includes peripheral immune organs such as the bone marrow, spleen, and blood (4). Patients with cancer exhibit a functionally altered immune macroenvironment that affects both distal organs and the tumor itself (5). Blood, which consists of plasma and various immune cells, is an important component of tumor immunity. The plasma portion of blood is an easily accessible biological sample, and changes in plasma protein expression can provide a basis for diagnosis (6). The expansion of peripheral T cells can also be used to predict tumor infiltration and clinical responses (7). During tumor progression, a patient’s immune cells exhibit a decline in tumor tissue infiltration ability (8); decreased immune infiltration is associated with poor disease control, whereas enhancements of peripheral immune cell function can improve tumor immunotherapy outcomes (9). The identification of changes in peripheral immunity can help to understand overall tumor immunity.

The immune system is a network. Various types of immune cells and communication among those cells are involved in the dynamic regulation of immunity (10). Tumor immunity is a complex process involving multiple organs and cells, including tumor cell growth *in situ*, tumor antigen presentation, peripheral immune cell activation, and tumor killing (11). Peripheral circulation is the final pathway by which activated immune cells infiltrate tumors; the dynamics of this pathway can reflect the systemic immune status. However, efforts to characterize immune status in CRC have mainly focused on the tumor microenvironment (12), liver metastases at the transcriptomic level (13), or the distributions of various immune cell subsets (14). To our knowledge, there is not a clear description of peripheral immune cells in CRC, and there has been minimal progress in immune cell-focused quantitative proteomic research with respect to CRC. An understanding of protein characteristics and dynamic alterations of the peripheral immune system in CRC would help to detect diagnostic indicators and identify new targets that can improve immunotherapy efficacy.

Here, we collected peripheral blood from patients with CRC and mapped their peripheral immune profiles at multiple levels, including protein expression dynamics, subpopulations of cells, and intercellular interactions. We identified characteristic changes in the peripheral immune system in CRC and then explored whether these features could serve as diagnostic indicators and therapeutic targets. The results of this study will contribute to a better understanding of the peripheral immune system in CRC and provide insights that facilitate the diagnosis and treatment of CRC.

## Experimental Procedures

### Experimental Design and Statistical Rationale

The first part of this study is the generation of the peripheral immune system proteomic datasets where we collected 33 samples for quantitative proteomic analysis of immune cells and 48 samples for quantitative proteomic analysis of plasma. By comparing protein expression differences between healthy donors and patients with CRC, we obtained characteristic proteins and explored the potential of these proteins for disease diagnosis and treatment by *in vitro* experiments or bioinformatics analysis. For immune cell subtypes analysis and cell–cell interaction analysis, we used the peripheral blood immune cell proteome dataset (10) or the receptor/ligand database as a reference to reveal alterations in immune cell proportion and receptor/ligand interactions by bioinformatics analysis. Finally, we explored the disease marker potential of plasma significantly differentiated proteins by algorithms such as random forests.

Proteomic analyses were conducted using packages in R software. The cleaned proteomic data were statistically analyzed using the *DESeq2* package (version 1.24.0) in R software. Proteins with statistically significant changes were selected using the criteria of adjusted *p*-value <0.05 and |Log_2_ fold change| >0.5. Pearson correlation analysis was performed using the cor function of the *stats* package (version 3.6.3) in R software. For phosphorylation proteomic data, peptide phosphorylation levels were calculated from phosphorylated peptide abundance and affiliated protein abundance. Student’s *t* test was used for the selection of phospho-peptides differentially expressed for *p* value <0.05 and |Log_2_ fold change| >0.5.

Statistical analysis of the other data was performed using GraphPad Prism 8.0 software. Results are expressed as the mean ± standard error of the mean. Comparisons between the two groups were performed using unpaired Student's *t*-tests. Welch’s correction was applied to adjust for differences in data distributions. *p*-values <0.05 were considered statistically significant. Other *p-*values were as follows: non-significant (ns) *p* > 0.05, ∗*p* < 0.05, ∗∗*p* < 0.01, ∗∗∗*p* < 0.001, and ∗∗∗∗*p* < 0.0001. All functional experiments were independently repeated at least three times.

### Clinical Samples

Peripheral blood samples were obtained from healthy donors and patients with CRC at Beijing Chao-Yang Hospital. Tumor grading was performed by clinicians, based on the histological characteristics of each tumor. In total, 33 samples were used for proteomic analysis of immune cells; 48 samples were used for proteomic analysis of plasma. The study protocol was reviewed and approved by the Chao-Yang Hospital Medical Ethics Committee (No. 2021-3-1-17). This study abides by the Declaration of Helsinki. All patients received an explanation of the study protocol and provided written informed consent for research analyses of their peripheral blood. Clinical information of the patients was de-identified, removing any personally identifiable information. The distribution of pathological characteristics of the samples involved in the experiment is shown in Supplemental Tables S1 and S2. Detailed pathology information for each donor is provided in Supplemental Table S5.

### Isolation of Plasma and Immune Cells

Blood from healthy donors and colorectal cancer patients was collected from Beijing Chao-Yang Hospital. Blood samples were collected using K2EDTA tubes (4 ml, BD Vacutainer) and transported to the laboratory at room temperature within 4 h of *ex vivo*. All blood samples were separated from plasma and a variety of blood immune cells on the same day of *ex vivo*.

Peripheral blood was centrifuged at 3000 rpm for 10 min to obtain plasma. Peripheral blood mononuclear cells were isolated using Ficoll-Paque Plus density gradients (GE Healthcare). Immunomagnetic beads (Miltenyi Biotec) were used to sort clusters of differentiation (CD)56^+^ natural killer (NK) cells, CD19^+^ B cells, CD8^+^ T cells, CD4^+^ T cells, and CD14^+^ monocytes. The purities of the sorted cells were detected by flow cytometry (FACSVerse, BD). The microbeads and antibodies used in this study are described in Supplemental Table S3.

Separated plasma samples were fractionated and stored at −80 °C until use. After magnetic sorting and purity testing, immune cells were immediately lysed with 1% SDS containing protease and phosphatase inhibitor cocktail (Beyotime) and stored at −20 °C until use. All samples underwent only one freeze-thaw cycle.

### Removal of High-Abundance Proteins From Plasma

The High Select Top14 abundant protein depletion mini spin column kit (Thermo Fisher Scientific) was used to remove high-abundance proteins from 10 μl aliquots of plasma. The top 14 abundant proteins removed include albumin, IgA, IgD, IgE, IgG, IgG (light chains), IgM, alpha-1-acid glycoprotein, alpha-1-antitrypsin, alpha-2-macroglobulin, apolipoprotein A1, fibrinogen, haptoglobin, and transferrin. Protein concentrations were measured with a bicinchoninic acid quantification kit (Thermo Fisher Scientific) before and after the removal of high-abundance proteins.

### Sample Preparation for Quantitative Proteomic and Phospho-Proteomic Analysis

For quantitative proteomic analysis, protein samples were precipitated with acetone at −20 °C overnight, resuspended in 8 M urea, and sonicated at 4 °C (Bioruptor). Alkylation was performed using dithiothreitol (5 mM) and iodoacetamide (15 mM). Protein samples were digested using LysC (1:100, w/w, Wako) and trypsin (1:50, w/w, Promega); the digestion was performed overnight at 37 °C with rotation at 200 rpm. The resulting mixtures were acidified and desalted using C18 StageTips (Empore); peptides were eluted using a refrigerated vacuum centrifuge. Next, peptides were labeled using a Tandem Mass Tag (TMT) Labeling Kit (Thermo Fisher Scientific). After labeling, the peptides were lyophilized and resuspended in double distilled water (pH = 10). Peptides were eluted with a 10% to 50% acetonitrile gradient, then lyophilized.

For shNC and sh-PTPRJ Jurkat cells, both label-free proteomic analysis and phosphorylated proteome analysis were performed. After enzymatic digestion, the mixtures were acidified and desalted using Oasis HLB solid phase extraction columns (Waters); peptides were eluted using a refrigerated vacuum centrifuge. After desalting, a small portion of peptides was analyzed by LC-MS/MS using an Orbitrap Exploris 240 mass spectrometer (Thermo Fisher Scientific), followed by label-free quantitative proteomic analysis. For phospho-proteomic profiling, phosphorylated peptides were enriched using the Fe-NTA phosphopeptide enrichment kit (Thermo Fisher Scientific) according to the instructions.

### Liquid Chromatography-Tandem Mass Spectrometry

For quantitative proteomic analysis, peptides were dissolved in 10 μl of 0.1% formic acid and separated on C18 columns by EASY-nLC 1200 nl liquid chromatography (Thermo Fisher Scientific). Chromatographic separation was performed using a linear gradient of 6% to 90% acetonitrile with 0.1% formic acid at a flow rate of 300 nl/min and a gradient time of 194 min. Mass spectrometry data were collected using an Orbitrap Fusion Lumos Tribrid mass spectrometer (Thermo Fisher Scientific). MS1 detection in the Orbitrap was conducted with a resolving power of 120,000. Tandem mass spectrometry identified 15 precursor ions with the strongest signal for high-energy collision dissociation (collision energy 37%), and MS2 detection in the Orbitrap was conducted with a resolving power of 50,000.

For label-free proteomic and phospho-proteomic analysis, peptides were dissolved in 10 μl of 0.1% formic acid and separated on C18 columns by EASY-nLC 1200 nl liquid chromatography (Thermo Fisher Scientific). Chromatographic separation was performed using a linear gradient of 0% to 80% acetonitrile with 0.1% formic acid at a flow rate of 300 nl/min and a gradient time of 120 min. Mass spectrometry data were collected using an Orbitrap Exploris 240 mass spectrometer (Thermo Fisher Scientific). MS1 detection in the Orbitrap was conducted with a resolving power of 120,000. Tandem mass spectrometry identified 15 precursor ions with the strongest signal for high-energy collision dissociation (collision energy 30%), and MS2 detection in the Orbitrap was conducted with a resolving power of 15,000.

### Analysis of Liquid Chromatography-Tandem Mass Spectrometry Data

Raw mass spectrometry data were analyzed using Proteome Discoverer software (version 2.2.0.388, Thermo Fisher Scientific), and tandem mass spectrometry spectra were searched against the human UniProt FASTA database (February 2019 edition, 95,556 entries) using SEQUEST-HT (Thermo Fisher Scientific). The enzyme was set to trypsin; the enzymatic specificity of trypsin was set to a maximum of 2 deleted cleavages and a minimum peptide length of 6 amino acids. Static modifications were set to carbamoyl methylation of cysteine (+57.021) and TMTpro (+229.163) linkage with lysine residues and peptide N termini. Variable modifications were set to acetylation of N termini (+42.011) and oxidation of methionine (+15.995). The total precursor ion mass tolerance was set to 10 ppm, and the product ion mass tolerance was set to 0.02 Da. A 1% false discovery rate threshold was applied at the peptide and protein levels. Peptides were normalized to the total peptide amount. Other parameters were set to default values. For label-free proteomic and phospho-proteomic data, Variable modifications were set to acetylation of N termini (+42.011), oxidation of methionine (+15.995), and phosphorylation of serine, threonine and tyrosine (+79.966). Other parameters were set to default values.

### Quality Control of Proteome Data

Samples from patients with CRC and healthy donors were distributed in different batches. Each batch contained a TMT-131-labeled mixed sample (mixture of all peptide samples) as the control sample for calibration across batches. Three sets of replicates were established for each batch of samples (three samples from each batch were prepared and detected twice by mass spectrometry). Water samples were used as blanks after every third injection to avoid carry-over. TMT labeling strategies are provided in Supplemental Table S5.

### Bioinformatics Analysis

For TMT experiments using 10 channels, 131 channels in each batch contained the same sample combination to facilitate data normalization. In this study, we excluded protein entries with missing values and erythrocyte contamination proteins (Supplemental Table S5); bioinformatics analysis was performed using proteins with quantitative data in all samples. The cleaned proteomic data exhibited a negative binomial distribution and were subjected to statistical analyses using the *DESeq2* package (version 1.24.0) (15) in R software. Proteins with statistically significant changes were selected using the criteria of adjusted *p* value <0.05 and |Log_2_ fold change| >0.5. For phosphorylation proteomic data, peptide phosphorylation levels were calculated from phosphorylated peptide abundance and affiliated protein abundance. Student’s *t* test was used for the selection of phospho-peptides differentially expressed for *p*-value <0.05 and |Log_2_ fold change| >0.5.

Metascape software (16) and the *clusterProfiler* package (version 3.12.0) (17) in R software were used for Gene Ontology annotation and Kyoto Encyclopedia of Genes and Genomes (KEGG) pathway analysis, respectively. The STRING database (http://string-db.org) (18) was utilized to identify protein networks; for subsequent analyses, we only selected interactions with experimental evidence and scored >0.4. Protein interactions were visualized using Cytoscape software (version 3.7.2) (19). Volcano plots and boxplots were constructed using the *ggplot2* package (version 3.3.2) in R software, and heatmaps were created using the *pheatmap* package (version 1.0.12) in R software.

### Analysis of Immune Cell Subtypes by Bioinformatics

Processed protein expression data were normalized using the *DESeq2* package (version 1.24.0) in R software. Reference peripheral immune cell proteomic data were obtained from a published article (10). In the reference proteome dataset, CD4^+^ T cells, CD8^+^ T cells, NK cells, B cells, and myeloid cells were divided into 15, 8, 8, 4, and 5 subsets, respectively. To quantify immune cell abundances in peripheral blood from patients with CRC, we used the analytical tool CIBERSORT (20) to estimate the proportions of cell types. The proportions of immune cells were separately predicted in each dataset. The proportions of immune cell subsets were compared between patients with CRC and healthy donors using two-sided Wilcoxon tests, and *p*-values <0.05 were considered statistically significant.

### Cell Culture and Transfection

Jurkat cells were cultured in RPMI 1640 (HyClone) supplemented with 10% fetal bovine serum (HyClone). PTPRJ expression was knocked down using the pLKO.1 vector. Stable PTPRJ-knockdown Jurkat cells were selected using 4 μg/ml puromycin (InvivoGen) for 3 days. Protein expression levels were evaluated by western blotting.

### Western Blotting

Proteins were denatured in SDS loading buffer by boiling for 10 min, separated by 7.5 to 12.5% SDS-PAGE, and transferred to PVDF membrane (Bio-Rad). Membranes were washed, blocked, and incubated with primary antibodies at 4 °C overnight. The following day, membranes were washed, and incubated with horseradish peroxidase-conjugated secondary antibodies, and protein signals were detected by ECL (Merck Millipore). Ponceau red was used to stain proteins in the PVDF membrane before blocking. Protein quantification was performed using ImageJ version 1.53r to analyze the intensity of protein bands or total protein. Relative expression of proteins was calculated by normalization with internal control (housekeeping protein expression (for cells) or total amount of protein (for plasma)). The antibodies used in this study are described in Supplemental Table S3.

### Reverse Transcription-Quantitative Polymerase Chain Reaction (RT-qPCR)

Total RNA was extracted using the EASYspin Plus RNA Mini Kit (Aidlab) and reverse-transcribed into single-stranded cDNA using the HiFiScript cDNA Synthesis Kit (CWBIO). RT-qPCR was performed with SYBR Green qPCR Master Mix (Promega). The reactions were conducted on a LightCycler96 SW 1.1 thermocycler (Roche). Expression levels were normalized to glyceraldehyde-3-phosphate dehydrogenase (*GAPDH*) in each sample and then standardized using the fold change metric. Primers used for RT-qPCR are listed in Supplemental Table S4.

### Flow Cytometry

For T cell subsets distribution validation, flow cytometry was performed on a BD FACSymphony A5 SE flow cytometer (BD Biosciences). The following monoclonal anti-human antibodies were used for cell sorting: CD45 (V500; clone HI30, Cat# 560779, BD), CD3 (Pacific Blue; clone OKT3, Cat# 317313, BioLegend), CD4 (APC/Cy7; clone RPA-T4, Cat# 300517, BioLegend), CD8 (FITC; clone HIT8a, Cat# 300905, BioLegend), CD45RA (APC; clone HI100, Cat# 304111, BioLegend) and CCR7(PE; clone G043H7; Cat# 353203, BioLegend).

For exploring the effects of PTPRJ on T cell function, flow cytometry was performed on a BD FACSVerse flow cytometer (BD Biosciences). The following monoclonal anti-human antibodies were used for cell sorting: interleukin (IL)-2-phycoerythrin (PE; clone MQ1-17H12, Cat# 500306, BioLegend), interferon (IFN)-γ-allophycocyanin (APC; clone 4S.B3, Cat# 502511, BioLegend), and CD25 (PE; clone BC96, Cat# 302605, BioLegend). Flow cytometry data analysis was performed using FlowJo software (BD Biosciences, version 10).

### Cell Proliferation Assays

Cell Counting Kit-8 (CCK-8) assay kits (Beyotime) were used, in accordance with the manufacturer’s instructions, to evaluate the effects of PTPRJ on Jurkat cells. Jurkat cells，shNC cells, and sh-PTPRJ cells were seeded in 96-well plates at a density of 5 × 10^3^ cells per well. Proliferation was measured every 24 h for 96 h. Cells were incubated with CCK-8 reagent (1:10) for 1 h before detection at each time point. A microplate reader (Multiskan FC, Thermo Fisher Scientific) was used to measure the absorbance at 450 nm.

### Ligand–Receptor Interaction Networks

Molecular definition files (for ligands and receptors) were downloaded from the Cell-Cell Interaction Database (https://baderlab.org/CellCellInteractions#ref1). Significantly changed proteins in each group were categorized as receptors, ligands, and others. Protein-protein interaction networks were constructed using the STRING online database (https://string-db.org/). Metascape software was used to exclude self-loops and ligand-ligand interactions, and then annotate ligand-receptor interactions.

### Plasma Biomarker Analysis

The *randomForest* package (version 4.6.14) in R software was used for random forest analyses with 10-fold cross-validation to achieve binary classification of patients with CRC and healthy donors. A random forest containing 500 trees was used to select the top 20 most important features according to mean accuracy reduction. The selected features were used for random forest analysis of an independent validation cohort. The *pROC* package in R software was used to calculate the area under the curve values and generate receiver operating characteristic curves.

### Analysis of Data From the Cancer Genome Atlas

The online database Gene Expression Profiling Interactive Analysis (GEPIA) (http://gepia.cancer-pku.cn/index.html) was used to confirm significant changes in plasma proteins. GEPIA (21) uses a standard processing pipeline that analyzes 275 tumors and 349 normal samples in the colon adenocarcinoma (COAD) dataset and 92 tumors and 318 normal samples in the rectum adenocarcinoma (READ) dataset; the data are collected from projects such as Genotype-Tissue Expression (GTEx) and The Cancer Genome Atlas (TCGA). Thresholds were |Log_2_ fold change| < 1 and *p* < 0.01. Other parameters were set to default values.

## Results

### Proteomic Analysis of Peripheral Immunity in CRC

**Fig. 1:**
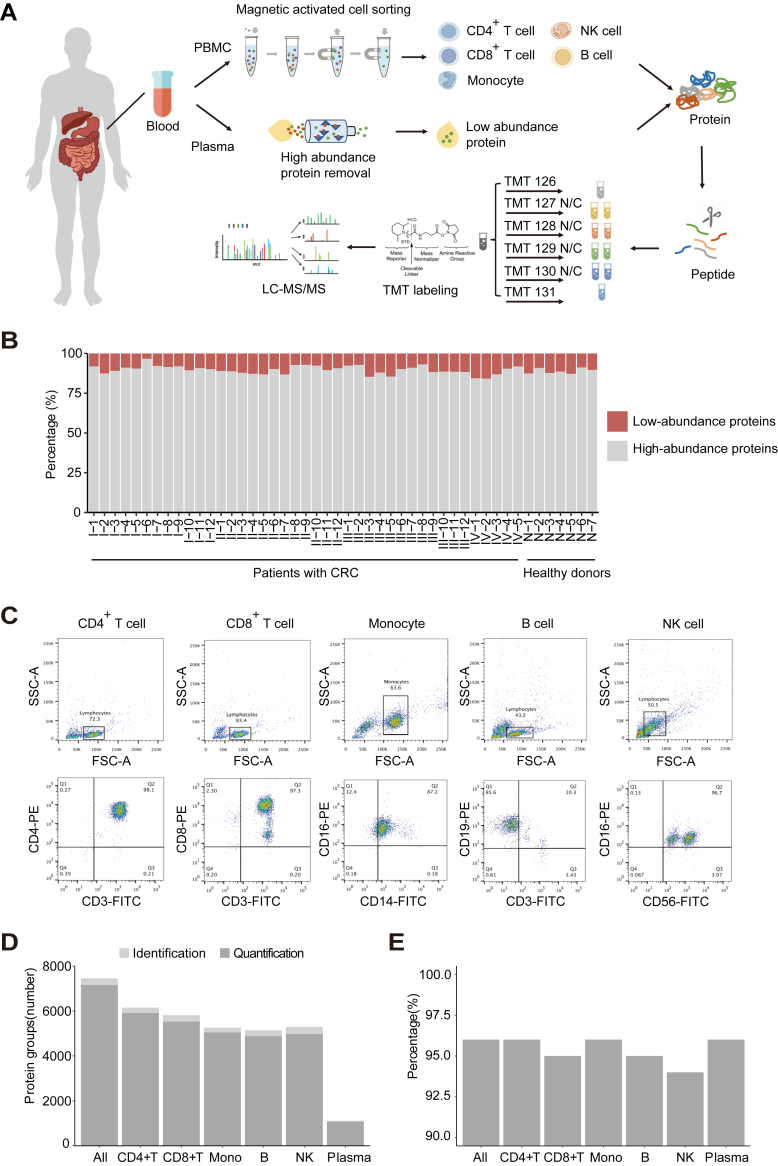
**Quantitative proteomic analysis of peripheral blood in colorectal cancer.***A*, schematic of experimental design and proteomic workflow. *B*, results of protein quantification by the BCA method, showing changes in the amount of plasma protein before and after removal of top14 high abundance protein. *C*, representative flow cytometry analysis of cells sorted by immunomagnetic beads. *D*, numbers of identified protein groups and quantified protein groups for each major cell lineage and plasma. CD4+T: CD4^+^ T cell, CD8+T: CD8^+^ T cell, Mono: monocyte, B: B cell, NK: NK cell. *E*, the ratio of quantified proteins to total identified proteins in each group.

For quantitative proteomic analysis, we isolated plasma from 48 donors (41 patients with CRC and 7 healthy donors), then sorted five types of immune cells from the peripheral blood of 33 donors (25 patients with CRC and 8 healthy donors) (Fig. 1*A*). The five types of immune cells included CD4^+^ T cells, CD8^+^ T cells, monocytes, B cells, and NK cells. For analysis of the plasma proteome, considering that high-abundance plasma proteins can hinder the identification and quantification of low-abundance proteins, we removed the top 14 high-abundance proteins, which constitute ∼90% of all plasma proteins (Fig. 1*B*). For analysis of the immune cell proteome, we used magnetic beads to sort immune cells and measured cell purity by flow cytometry; we then conducted mass spectrometry analysis using whole-cell lysates (Fig. 1*C*).

Peptide samples were labeled with 10-plex TMTs and analyzed by high-resolution mass spectrometry using an Orbitrap Fusion Lumos instrument. Replicate samples were analyzed in parallel to confirm experimental repeatability and instrumental stability. Three sets of replicates were established for each group of samples (Supplemental Table S5); correlation analysis between replicate samples showed that the overall experimental repeatability was good (*R*^2^ > 0.9), and the data exhibited high reliability (Supplemental Figs. S1 and S2, *A* and *B*). Using a peptide and protein false discovery rate of 1%, 1117 proteins were identified in the plasma proteome; 1074 proteins were quantified (∼96% of all identified proteins). In total, 7787 proteins were identified in the immune cell proteome; 7307 proteins were quantified (∼94% of all identified proteins) (Fig. 1, *D* and *E*).

### Proteomic Atlas of Plasma in CRC

**Fig. 2:**
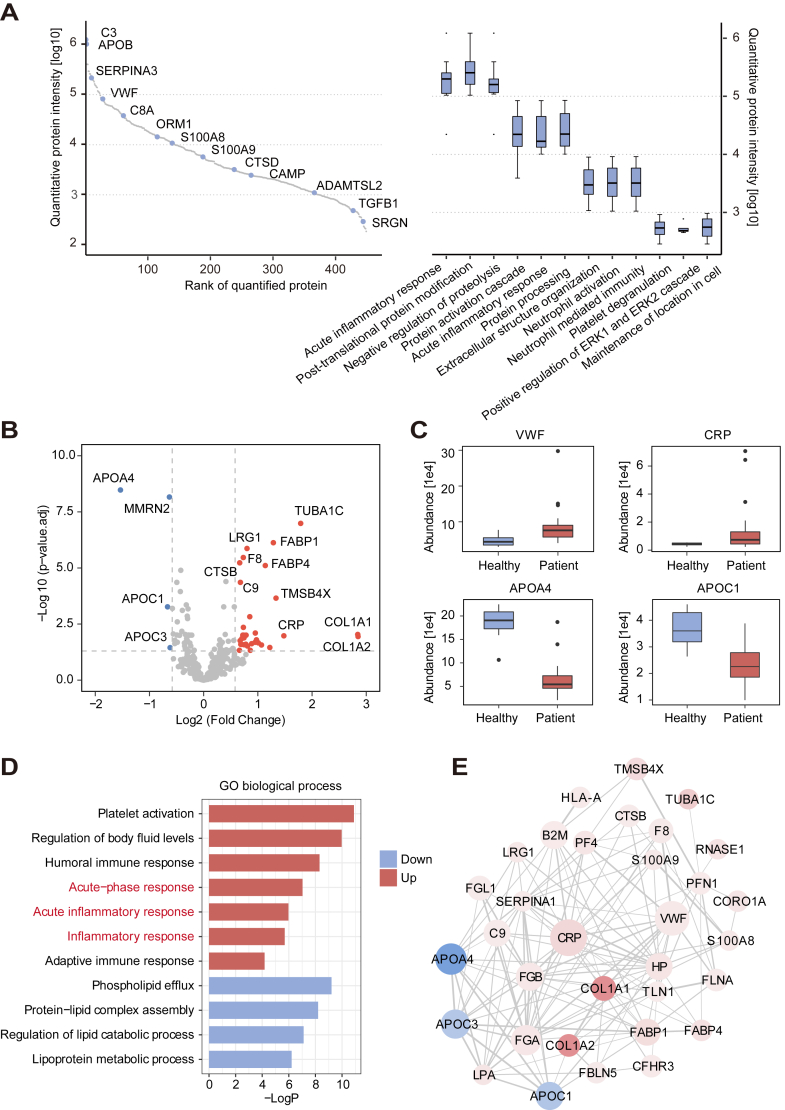
**Proteomic analysis of plasma in colorectal cancer.***A*, distribution of plasma protein abundances and annotation of biological processes. The 449 plasma proteins are ranked according to abundance (*left*), with a few representative proteins highlighted (*blue*). Biological process enrichment of proteins is shown according to abundance (*right*). *B*, significantly changed proteins in plasma from patients with colorectal cancer (CRC). *Red* represents significantly upregulated proteins, and blue represents significantly downregulated proteins. *C*, boxplots showing expression levels of VWF, CRP, APOA4, and APOC1 in plasma from patients with CRC (CRC) and healthy donors (HD). *D*, biological process enrichment analysis of significantly changed proteins in plasma. *Red* represents significantly upregulated proteins, and blue represents significantly downregulated proteins. *E*, protein–protein interactions of 37 significantly changed proteins in plasma. *Red* represents upregulated proteins, blue represents downregulated proteins, and the thickness of the *gray line* represents the interaction score.

For subsequent analysis, we selected 449 plasma proteins that were quantified in all samples. Plasma protein expression was measured by abundance values. The most highly expressed protein was complement C3, followed by APOB. Proteins were ranked according to abundance, then annotated according to function using Gene Ontology biological process terms. The results showed that acute-phase inflammatory response, negative regulation of protein degradation, and protein post-translational modification processes were enriched in high-abundance proteins; extracellular structure organization, neutrophil-mediated immunity, and platelet degranulation processes were enriched in low-abundance proteins (Fig. 2*A*).

To eliminate the effects of erythrocyte contamination during plasma collection, we removed 30 common erythrocyte-contaminating proteins (22) before analyzing them (Supplemental Table S5). Compared with plasma from healthy donors, there were 37 significantly changed proteins in plasma from patients with CRC: 33 were upregulated and 4 were downregulated (Fig. 2*B*, Supplemental Table S5). Immunoblotting results demonstrated high expression of plasma LRG1 and low expression of APOA4 in colorectal cancer, further validating the results of plasma quantitative proteomics (Supplemental Fig. S2, *C*–*F*).

The most upregulated protein was COL1A1(7.2-fold), and the most downregulated protein was APOA4 (2.9-fold). Typical acute-phase inflammatory plasma proteins such as CRP and VWF were upregulated by 2.8-fold and 1.7-fold, respectively. CRP (23), the most common indicator of systemic inflammatory response, is involved in tissue damage; its upregulation indicates the presence of inflammation in patients with CRC (Fig. 2*C*). In addition to acute-phase inflammatory processes, plasma differential proteins were enriched in platelet activation, regulation of body fluid levels, and other processes (Fig. 2*D*). There were close interactions among the differential proteins; proteins that exhibited more interactions were CRP, APOA4 and FGA (Fig. 2*E*). Plasma quantitative proteomic analysis suggested the presence of increased inflammation in patients with CRC.

### Proteomic Atlas of Peripheral Immune Cells in CRC

**Fig. 3:**
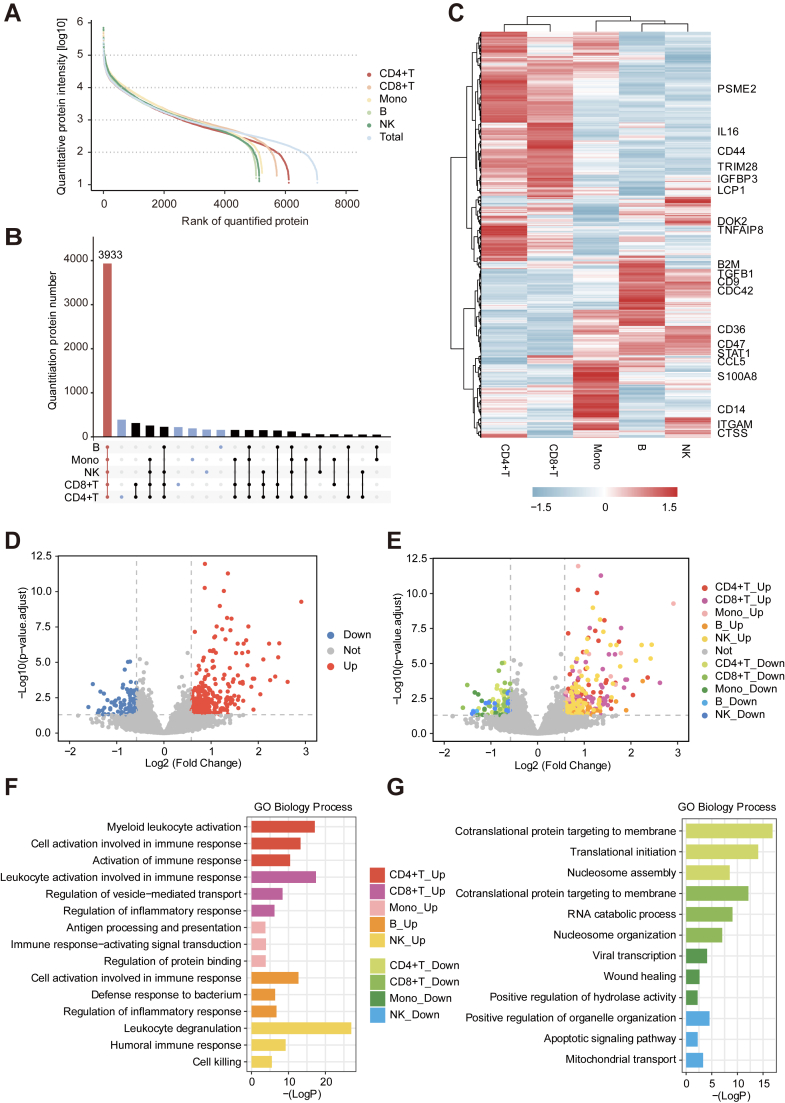
**Proteomic analysis of immune cells in colorectal cancer.***A*, dynamic range of protein abundance among measured cell lineages, with colors representing cell types. *B*, intersection of five cellular proteomes. *Red* represents the number of proteins in the five cellular proteomes, *blue* represents the number of unique proteins in the five cellular proteomes, and black represents the number of proteins in other cases. *C*, heatmap of 1623 proteins expressed in all 5 cell lineages. Row-column clustering was performed using Ward's method, and named proteins are representative of the indicated clusters. *D*, volcano plot showing differentially expressed proteins in patients with colorectal cancer and healthy donors. *Red* represents significantly upregulated proteins, and blue represents significantly downregulated proteins. *E*, comparison of blood cell proteomes between patients with colorectal cancer and healthy donors. *Colors* represent significant changes in protein expression and cellular origin. *F*, biological process enrichment analysis of significantly upregulated proteins in each cell lineage. *G*, biological process enrichment analysis of significantly downregulated proteins in each cell lineage.

For proteomic characterization of the peripheral immune system, we identified and quantified proteins from five types of immune cells (Fig. 3*A*). In total, 3933 proteins were expressed in all five types of immune cells (*i.e.*, shared) (Fig. 3*B*). By calculating the relative expression levels of the 1623 shared proteins quantitatively expressed in all samples, the expression patterns of the proteins were found to be cell lineage-specific (Fig. 3*C*). To eliminate the effects of erythrocyte contamination during plasma collection, we removed 30 common erythrocyte contaminating proteins (22) before analyzing (Supplemental Table S5). Compared with healthy donors, there were 456 significantly changed proteins across the five types of immune cells in patients with CRC: 357 were upregulated and 99 were downregulated (Fig. 3*D*, Supplemental Table S5). The numbers of significantly changed proteins in CD4^+^ T cells, CD8^+^ T cells, monocytes, B cells and NK cells were 163, 127, 41, 23, and 102, respectively (Fig. 3*E*). Most upregulated proteins were associated with the immune system processes (Fig. 3*F*). In contrast, downregulated proteins were associated with biological processes such as nucleosome assembly, membrane protein localization, and regulation of cell adhesion (Fig. 3*G*).

Next, we performed further analysis for significantly changed proteins for each type of immune cell. Among CD4^+^ T cells, the expression of T cell activation proteins(CD3ζ, FCGR2A, CD84 (24, 25, 26, 27)) and IFN-related proteins (WDFY1, IRF3 (28, 29)) were upregulated. Our data showed significant upregulation of the expression levels of protein tyrosine phosphatases (*e.g.*, PTPRA, PTPRE, PTPRJ, and DUSP3) with roles in the TCR signaling pathway. The high expression levels of these regulators may inhibit the phosphorylation of key proteins downstream of the TCR pathway (Supplemental Fig. S3*A*). CD8^+^ T cells displayed an immune activation phenotype, mainly characterized by high expression levels of HLA-F (30), FCGR2A, and CD7 (31, 32). Additionally, CD8^+^ T cells showed high expression levels of proteins associated with cell migration and adhesion (*e.g.*, CCR2, CD49b, MMP8, and MMP9) (33, 34) (Supplemental Fig. S3*B*). For monocytes, the expression of cell adhesion regulators such as CD64, VNN2 (35), CD9 (36), ARRB2, and STX6 (37) were upregulated (Supplemental Fig. S3*C*). Notably, inflammation-related proteins (RIPK1, STING, and FCGR1A) were upregulated (38, 39, 40). Among B cells, the expression levels of HLA-A, HLA-DMA, S100P, S100A8, and S100A9 were significantly upregulated (Supplemental Fig. S4*A*). In patients with CRC, NK cells showed functional activation and increased expression of cathepsin family proteins. Intracellular expression levels of CTSB, CTSS, CTSG (41), and CTSH (42) were significantly increased; the significant decrease in CTSW (43) implied alterations in cell activation (44) (Supplemental Fig. S4*B*). These results suggest that protein expression in the peripheral immune system is altered in patients with CRC during disease progression.

### Distribution Analysis of Peripheral Immune Cell Subpopulations

**Fig. 4:**
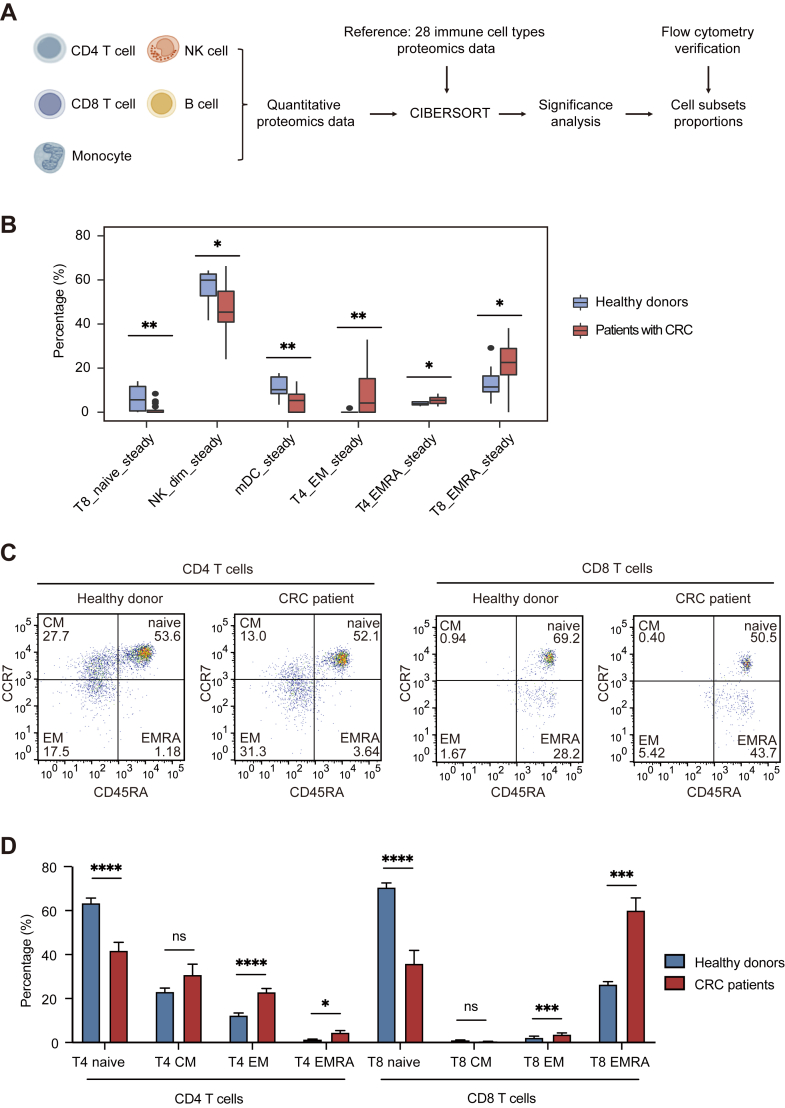
**Distribution of peripheral immune cell subtypes.***A*, schematic of the workflow for analyzing and validating the distribution of immune cell subpopulations. *B*, bioinformatics analysis results reveal significantly changed cell subsets in patients with colorectal cancer. The Wilcoxon test was used to compare cell subsets between healthy donors and patients with cancer and to identify significantly altered cell subsets (*p*-value <0.05). Non-significant (ns) *p* > 0.05, ∗*p* < 0.05, ∗∗*p* < 0.01, ∗∗∗*p* < 0.001, ∗∗∗∗*p* < 0.0001. *C*, representative flow cytometry analysis of peripheral T cell subsets (T_naive_, T_CM_, T_EM_, T_EMRA_) in colorectal cancer patients and healthy donors. *D*, statistical graph of changes in the distribution of T cell subsets (n = 3 per group; mean ± standard error of the mean, Welch's *t* test). Non-significant (ns) *p* > 0.05, ∗*p* < 0.05, ∗∗*p* < 0.01, ∗∗∗*p* < 0.001, ∗∗∗∗*p* < 0.0001.

Many immune cells exhibit multiple subtypes with distinct functions. In this study, the human peripheral blood immune cells proteome served as the reference dataset (10); quantitative proteomic data from five types of immune cells were analyzed by using CIBERSORT (45) to classify cell subtypes (Fig. 4*A*). This classification approach revealed that the five major types of immune cells could be categorized into 7, 6, 8, 4, and 5 subtypes (Supplemental Fig. S4*C*). The distributions of the 6 cell subtypes were significantly altered in patients with CRC relative to healthy donors, in whom all cells exhibited a steady-state distribution (Fig. 4*B*). Specifically, the proportions of naïve CD8^+^ T cells (T8_naive_steady), CD56^dim^ NK cells (NK_dim_steady), and myeloid dendritic cells (mDC_steady) were significantly decreased in patients with CRC. Conversely, the proportions of effector memory CD4^+^ T cells (T4_EM_steady), effector memory CD45RA^+^ CD4^+^ T cells (T4_EMRA_steady), and effector memory CD45RA^+^ CD8^+^ T cells (T8_EMRA_steady) were significantly increased.

Further, we validated the results by flow cytometry for the cell subpopulations classified by CIBERSORT (Supplemental Fig. S5). Colorectal cancer patients exhibit a decrease in peripheral CD4^+^ and CD8^+^ naive T cells(CD45RA^high^, CCR7^high^). The proportions of effector T cells(CD45RA^low^, CCR7^low^) and terminally-differentiated effector cells(CD45RA^high^, CCR7^low^) subsets were increased in both CD4^+^ and CD8^+^ T cells (Fig. 4, *C* and *D*). These findings suggest that effector memory T cells are increased in patients with CRC, reflecting the immune system's response to tumor signals.

### Changes in Ligand–Receptor Interaction Dynamics

**Fig. 5:**
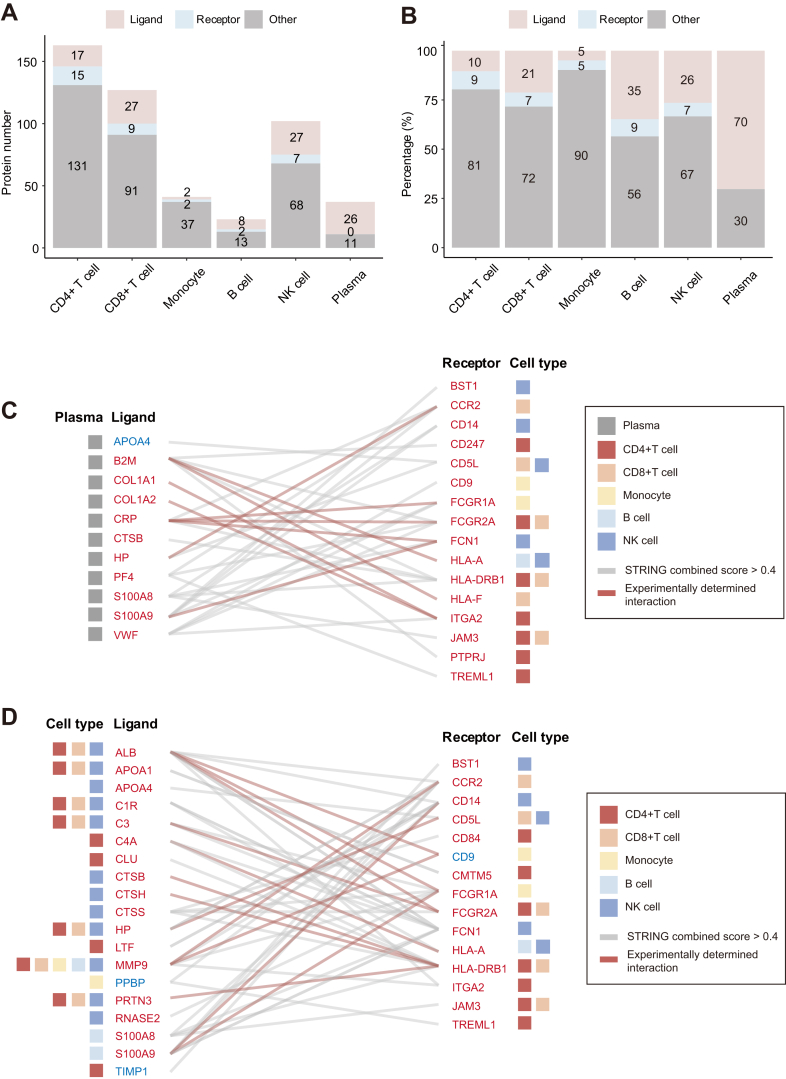
**Changes in ligand-receptor interactions in peripheral immunity.***A*, numbers of receptor and ligand proteins among significantly changed proteins in each group. *B*, proportions of receptor and ligand proteins among significantly changed proteins in each group. *C*, interactions of plasma-derived ligands with cell-surface receptors. *D*, interactions of ligands and receptors among various immune cells. The Colors indicate cell types from which the proteins are derived; connecting lines indicate interactions between receptors and ligands, where a *gray line* represents a STRING score >0.4, and a *red line* represents an experimentally proven interaction; protein names in red indicate significant upregulation, and protein names in blue indicate significant downregulation.

Immune system functionality depends on communication among multiple types of cells. To identify altered mechanisms of communication (46) in CRC, we categorized the significantly changed proteins in immune cells and plasma into ligands, receptors, and others (Fig. 5*A*). Among the 37 significantly changed proteins in plasma, there were 26 ligands and no receptors. B cells had the largest proportion of ligands, comprising ∼30% of the total (Fig. 5*B*).

To further characterize the communication structure within the immune system, we used the STRING database to analyze interactions among 26 ligand proteins in plasma and 35 receptor proteins expressed by five types of immune cells (Fig. 5*C*). The biological process enrichment results suggested that the interactions of plasma-derived ligands with immune cell-surface receptors play roles in IFN-γ-related pathways, immune cell migration, inflammatory responses, and cytokine secretion. Notably, there were 11 experimentally proven interactions, including COL1A1-ITGA2, COL1A2-ITGA2-mediated cell activation (47), CRP-FCGR2A-mediated cellular inflammatory response (48), and S100A9-FCN1-mediated immune defense.

Next, we characterized ligand-receptor interactions among the five types of immune cells. We used the STRING database to analyze interactions among 81 ligands and 35 receptors expressed by the five types of immune cells (Fig. 5*D*). The biological process enrichment results suggested that the interactions between ligands and receptors play roles in multiple biological processes, including complement system activation (C1R-FCN1), inflammatory response (CLU-SORL1, LTF-CD14, HP-CCR2), cell migration (MMP9-CCR2), and immune defense regulation (APOA1-CMTM5). Our analysis of ligand-receptor interactions revealed changes in peripheral immune system dynamics in CRC. It should be noted that the above analyses are all hypothetical results based on database annotations and analyzed by bioinformatics, which still need further experimental verification.

### Protein Characterization During CRC Progression

**Fig. 6:**
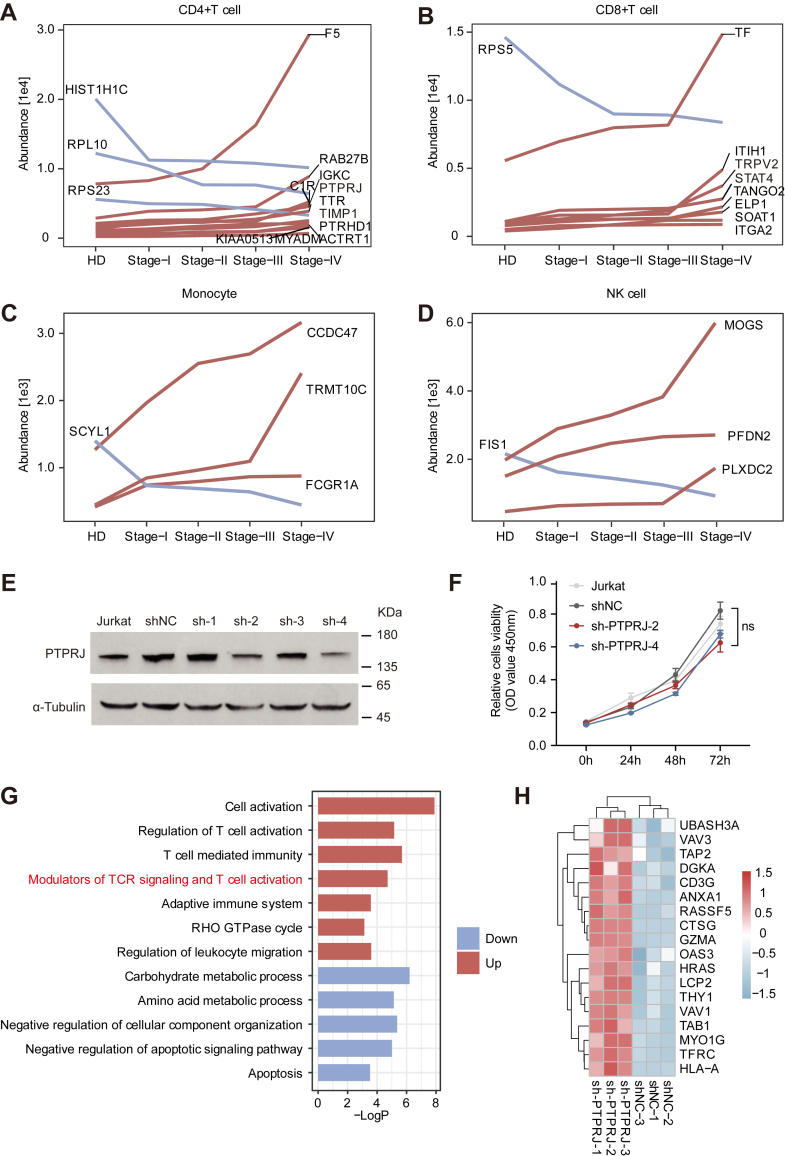
**Trends in peripheral immune protein expression.***A*, relative expression levels of significantly changed proteins in CD4^+^ T cells at various stages of colorectal cancer (CRC). *Red* represents proteins with increasing expression, and *blue* represents proteins with decreasing expression. *B*, relative expression levels of significantly changed proteins in CD8^+^ T cells at various stages of CRC. *C*, relative expression levels of significantly changed proteins in monocytes at various stages of CRC. *D*, relative expression levels of significantly changed proteins in NK cells at various stages of CRC. *E*, Western blotting analysis of the extent of PTPRJ knockdown in Jurkat cells. *F*, PTPRJ knockdown does not affect T cell proliferation (n = 4 per group; mean ± standard error of the mean, Student’s *t* test). Non-significant (ns) *p* > 0.05, ∗*p* < 0.05, ∗∗*p* < 0.01, ∗∗∗*p* < 0.001, ∗∗∗∗*p* < 0.0001. *G*, biological process enrichment analysis of significantly changed proteins in sh-PTPRJ cells. *H*, heatmap of protein expression associated with the TCR signaling pathway in significantly changed proteins of sh-PTPRJ cells.

Proteins that exhibit changes according to disease progression are potential therapeutic targets for enhanced peripheral immune function in CRC. In CD4^+^ T cells, 11 proteins were elevated with disease progression and 3 proteins gradually decreased. Notably, the level of PTPRJ expression in CD4^+^ T cells gradually increased with CRC progression (Fig. 6*A*), suggesting that the TCR signaling pathway might be inhibited (49). In CD8^+^T cells, the expression levels of RPV2 and STAT4 were elevated with disease progression, suggesting that cell activation was enhanced with the increase of tumor-associated signals (Fig. 6*B*). The expression levels of CCDC47, TRMT10C, and FCGR1A in monocytes increased with disease progression, whereas the level of SCYL1 decreased. The level of FCGR1A was positively correlated with inflammation (50), suggesting that monocyte-associated inflammatory responses were enhanced in patients with cancer during disease progression (Fig. 6*C*). The expression levels of MOGS, PFDN2, and PLXDC2 in NK cells increased with disease progression, whereas the expression of FIS1 decreased (Fig. 6*D*). Low expression of FIS1 is associated with mitophagy and impaired mitochondrial respiration (51). These results suggest that protein expression in the peripheral immune system is altered in patients with CRC during disease progression. These proteins with altered trends have potential as markers and therapeutic targets.

Further, we hope to demonstrate the therapeutic potential of these proteins that change with disease progression through *in vitro* experiments. Considering that CD4^+^ T cells play a key role in tumor immunity and that the inhibition of TCR signaling may affect anti-tumor activity in CD4^+^ T cells (52), we explored whether PTPRJ could serve as a therapeutic target for enhanced peripheral immunity. To determine whether PTPRJ had a regulatory effect on CD4^+^ T cell function, we constructed PTPRJ-knockdown Jurkat cells (Fig. 6*E*). Knockdown of PTPRJ did not affect T cell proliferation (Fig. 6*F*) but elevated the expression level and phosphorylation level of TCR signaling pathway-related proteins. In particular, the phosphorylation levels of LCK (S94, Y192, S194) and PLCγ (S1233, S1236) were elevated (Fig. 6, *G* and *H* and Supplemental Fig. S6, *A*–*C*). These results demonstrate that PTPRJ can affect the activation of the TCR signaling pathway. Under the same conditions, the level of activation was elevated among PTPRJ-knockdown T cells, and the cell-surface expression level of CD25 was upregulated by approximately 1.5-fold (Supplemental Fig. S6*D*). Moreover, the levels of IL-2 and IFN-γ secretion were increased. Upon stimulation with phytohemagglutinin (PHA), IL-2 secretion increased from 1.62% to 7.24% after PTPRJ knockdown (4.5-fold upregulation), and IFN-γ secretion increased from 0.041% to 0.14% (3.4-fold upregulation) (Supplemental Fig. S6, *E* and *G*). These results were confirmed by RT-qPCR assays, which showed that the expression levels of *IL-2* and *IFN-γ* were increased in PTPRJ-knockdown Jurkat cells under steady-state and activated conditions (Supplemental Fig. S6, *F* and *H*). These results suggest that the high expression of PTPRJ in patients with CRC may cause CD4^+^ T cells to exhibit immunosuppression. PTPRJ inhibition is expected to increase the levels of T cell activation and cytokine secretion.

### Screening of Potential Plasma Biomarkers for Colorectal Cancer

**Fig. 7:**
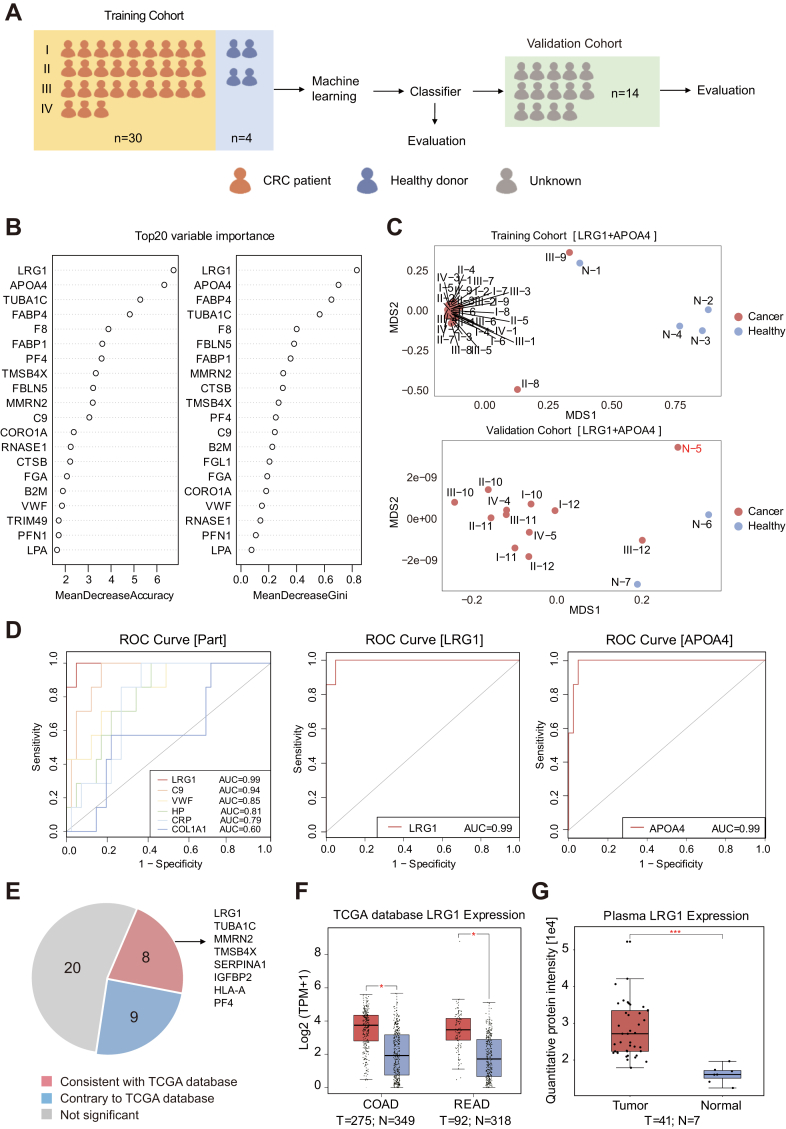
**Machine learning-based identification of plasma markers for colorectal cancer.***A*, study design for machine learning-based identification of plasma biomarkers for colorectal cancer (CRC). Samples were obtained from a training cohort (n = 34) for proteomic analysis. Potential plasma biomarkers were tested in an independent validation cohort (n = 14). *B*, top 20 most important plasma proteins, ranked according to mean decrease in accuracy and mean decrease in Gini. *C*, model performances (LRG1+APOA4) in the training and validation cohorts. Samples colored in red indicate incorrect identification as patients with CRC. *D*, receiver–operating characteristic (ROC) curves of significantly changed proteins in plasma. *E*, venn diagrams show that 8 significantly changed proteins in plasma have the same expression trends in tumors *in situ*. *F*, boxplots show that LRG1 expression was significantly upregulated in CRC at the transcriptomic level. *G*, boxplots showing the expression levels of LRG1 in plasma from patients with CRC (CRC, n = 41) and healthy donors (HD, n = 7).

The current clinical diagnostic paradigm for CRC typically uses fecal occult blood testing as a pre-screening method, followed by colonoscopy (53). The optimal diagnostic modality should be non-invasive, and peripheral blood is an easily accessible biosample that can be used for tumor screening. We used machine learning methods to explore potential plasma biomarkers. Thirty-four samples were used as the training cohort to identify potential biomarkers, and 14 samples were used as the validation cohort to test the identified biomarkers (Fig. 7*A*). In the training cohort, binary classification to distinguish between healthy donors and patients with CRC was conducted using a random forest that contained 500 trees; the top 20 most important features were selected for further analysis (Fig. 7*B*). These selected features were used to perform random forest analysis in the independent validation cohort. The highest-ranked protein was LRG1, followed by APOA4 and TUBA1C. The results showed that LRG1+APOA4 was the best combination for distinguishing patients with CRC from healthy donors; its accuracies were 100% in the training cohort and 92.85% in the validation cohort (Fig. 7*C*). Next, receiver–operating characteristic analysis was performed and area under the curve values were calculated for 37 significantly changed proteins. LRG1 and APOA4 had the highest area under the curve values (both 0.99) (Fig. 7*D*).

Finally, we searched the TCGA database to determine the expression patterns of 37 plasma proteins at the transcriptome level. The results showed that 9 proteins had the same expression trend in the transcriptome, 9 had the opposite trend, and the remaining 20 proteins did not exhibit a significant change (Fig. 7*E*). LRG1 was significantly elevated in the *in situ* tumor transcriptome data (Fig. 7*F*), consistent with the findings in plasma (Fig. 7*G*). Taken together, these results suggest that LRG1 can serve as a single-protein biomarker for distinguishing between patients with CRC and healthy donors, whereas LRG1+APOA4 constitutes a protein combination that can characterize disease status. Using a machine learning approach, we determined that the combination of LRG1 and APOA4 serves as the most effective predictive plasma marker for colorectal cancer. However, it is crucial to note that the accuracy of the predictions is influenced by the composition of the sample cohort. Furthermore, it is essential to validate the accuracy and sensitivity of LRG1 and APOA4 in larger sample cohorts, including those from patients with various types of cancer.

## Discussion

Immune responses are key regulators of tumor progression. Tumors exhibit type-specific responses to immunotherapy, which indicates that tumor immunity is heterogeneous and complex. Therefore, a more comprehensive framework is needed to understand the tumor-associated changes in the immune system. In recent years, studies have begun to focus on the effects of systemic immunity on tumor suppression (9); however, few reports have described the peripheral immune system at the proteomic level. The present study provided a quantitative proteomic profile of plasma and five types of immune cells, with detailed characteristics of peripheral immunity and supporting data necessary to understand functional alterations of peripheral immunity in CRC.

An excessive inflammatory response can contribute to CRC onset (54). Our analysis of quantitative proteomic data revealed that the upregulation of inflammation-related proteins is a primary characteristic of CRC. This is mainly manifested by increased expression of CRP in plasma, increased expression of RIPK1 and STING (associated with cellular inflammatory signaling) in monocytes, and increased levels of ligand-receptor interactions associated with inflammation. Currently, nonsteroidal anti-inflammatory drugs (*e.g.*, aspirin) exhibit chemopreventive potential against CRC (55). The present study showed that the inflammatory activation of peripheral monocytes can exacerbate the inflammatory response *in vivo*. Therefore, targeted inhibition of monocyte inflammatory pathways with excess activation may provide new ideas for alleviating the inflammatory response in CRC.

Blood is an important aspect of tumor immunity. Functionally, it is the final route by which activated immune cells can enter the tumor microenvironment. Our data reveal that the levels of peripheral immune cell activation are higher in patients with CRC compared to healthy donors. Particularly among CD8^+^ T cells, the level of STAT4 expression gradually increased with disease progression. Evidence suggests that type I IFNs enhance the cytotoxicity of human CD8^+^ T cells *via* STAT4-and granzyme B-dependent pathways (56). The IL-12/STAT4/T-bet axis plays an important role in CD8^+^ effector T cell responses (57). In contrast to the findings in CD8^+^ T cells, peripheral CD4^+^ T cells showed functional alterations that were detrimental to tumor suppression. During disease progression, the severity of TCR pathway inhibition increases. Elevated expression of PTPRJ inhibits TCR-mediated activation (49). Elevated expression levels of SNX27 and GRAP inhibit ZAP-70 function after TCR activation (58, 59). DUSP3, a target of ZAP-70, inhibits Erk2 pathway activation in a manner that regulates ZAP-70 (60, 61). There is evidence that TCR diversity among peripheral circulating T cells is reduced in breast cancer (62), lung cancer (63), and CRC (64); these decreases in diversity are associated with poor tumor control. The present study revealed the phenotype of peripheral CD4^+^ T cells with TCR pathway suppression among patients with CRC. *In vitro* experiments identified PTPRJ as a potential therapeutic target for enhanced peripheral immunity.

The distribution of immune cell subpopulations is a critical indicator of immune status. Advances in single-cell technology have enabled CRC tumor tissue and liver metastases to undergo comprehensive single-cell transcriptomic analyses; tumor-infiltrating T cells (65), innate lymphocytes (66), granulocytes (13), stromal cells (67), and myeloid cells (68) have been extensively described. The advent of single-cell mass spectrometry has facilitated the investigation of cellular subpopulations at the protein level. The innate lymphocyte population (Lin^−^CD7^+^CD127^−^CD56^+^CD45RO^+^) is reportedly enriched in CRC tissue and displays cytotoxic activity, indicating the existence of tumor-resident innate and adaptive immune cell populations (14). In this study, we categorized cell subpopulations using quantitative proteomic data and bioinformatics analysis and subsequently validated the results by flow cytometry. We found that the proportions of effector memory CD4^+^ T cells, effector memory CD45RA^+^ CD4^+^ T cells, and effector memory CD45RA^+^ CD8^+^ T cells were increased in CRC, reflecting active immune responses to tumor signals. However, the proportions of naive CD8^+^ T cells, CD56^dim^ NK cells, and myeloid dendritic cells were reduced, indicating the potential for decreased T cell activation and lower tumor cell-killing capacity. Our results provide insights into changes in peripheral immune cell subsets that occur in CRC.

The identification of plasma biomarkers that can reflect disease status has potential clinical value. Multiple analysis methods indicate that LRG1 may be a useful single plasma biomarker for CRC, while the combination of APOA4 and LRG1 may serve as a good indicator. The potential of LRG1 as a tumor biomarker has also been demonstrated in other studies (69, 70). Bhardwaj *et al* (71) investigated the potential clinical value of 175 serum protein markers of CRC in a training set of 200 participants (100 patients with CRC and 100 healthy controls) and a validation set of 155 participants (56 patients with CRC and 99 healthy controls). The overall ranking of LRG1 in the validation set was high. Our findings include a library of plasma protein biomarkers that can discriminate among various pathological conditions and have potential clinical value. However, further validation should be performed in studies with larger numbers of patients and the specificity of the biomarkers should be verified in multiple diseases.

In conclusion, the present study provides a quantitative proteomic profile of the peripheral immune system in CRC, with a detailed exploration of the tumor-associated functional alterations to peripheral immunity regarding protein expression, cell subtypes, and ligand-receptor interactions. The results showed phenotypes that are detrimental to tumor suppression, such as enhanced inflammatory responses, suppression of the TCR signaling pathway, and reduced populations of immune cells with tumor cell-killing capacity. Additionally, we explored the potential applications of these protein characteristics in areas such as enhancing peripheral immunity and improving tumor diagnosis. Our results support a comprehensive understanding of tumor peripheral immunity and offer new options for the diagnosis and treatment of CRC.

## Data Availability

Proteomic datasets generated and/or analyzed in this study have been deposited to the ProteomeXchange Consortium (http://proteomecentral.proteomexchange.org) *via* the iProX partner repository with the dataset identifier PXD046254. The significantly changed proteins are included in Supplemental Table S5.

## Supplemental data

This article contains supplemental data.

## Conflict of interest

The authors declare that they have no known competing financial interests or personal relationships that could have appeared to influence the work reported in this paper.
