# Supplementary material for: Quantitative Proteomic Analysis Reveals Functional Alterations of the Peripheral Immune System in Colorectal Cancer
Source: Mol Cell Proteomics. 2024 May 11;23(6):100784. doi: 10.1016/j.mcpro.2024.100784 (PMC11215959; doi:10.1016/j.mcpro.2024.100784)
Supplement: Supplemental Materials [file mmc1.docx]

**Quantitative proteomic analysis reveals functional alterations of the peripheral immune system in colorectal cancer**

Wenyuan Zhu^1,2^, Minzhe Li^3^, Qingsong Wang^1,2,*^, Jian Shen^3,*^, Jianguo Ji^1,2,*^

^1^State Key Laboratory of Protein and Plant Gene Research, School of Life Sciences, Peking University, Beijing, China.

^2^Department of Biochemistry and Molecular Biology, School of Life Sciences, Peking University, Beijing, China.

^3^General Surgery Department, Beijing Chao-Yang Hospital, Capital Medical University, Beijing, China.

**Supplemental materials**
1, Supplemental Figures S1-6

2, Supplemental Tables S1-4.

**
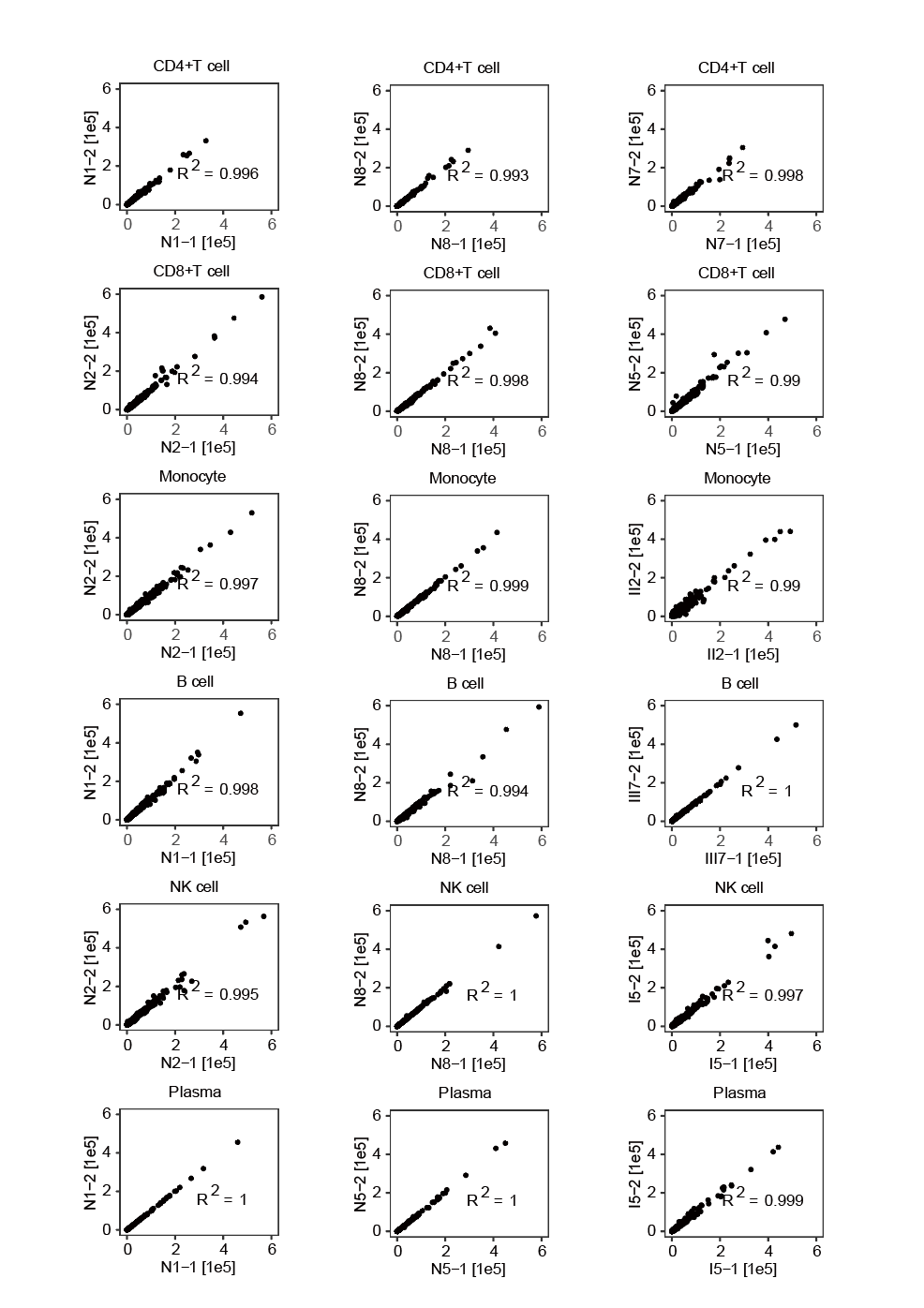
**

**Supplementary Figure 1. Quantitative proteomic data quality control.**

Pearson correlation analysis among replicate samples of blood component proteomes. Three replicate samples were established for analyses of the plasma proteome and the five immune cell proteomes.


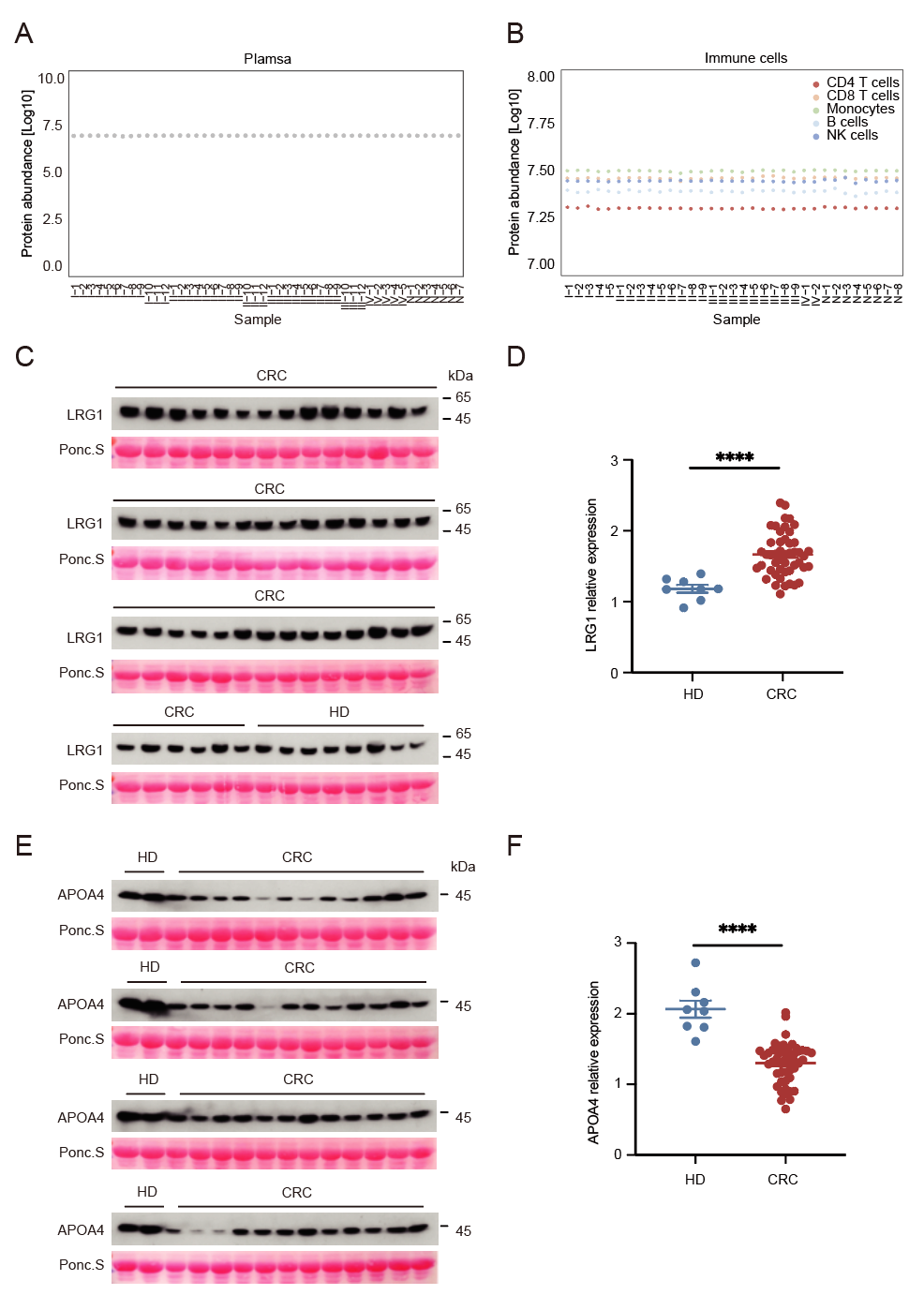


**Supplementary Figure 2. Protein abundance distribution of mass spectrometry data between samples.**

(A) Plasma protein abundance distribution of mass spectrometry data from different samples for bioinformatics analysis. (B) Distribution of abundance of five types of immune cell proteins in mass spectrometry data from different samples for bioinformatics analysis. (C) Western blotting analysis of LRG1 expression in plasma from CRC patients and healthy donors. (D) Quantification analysis of LRG1 in plasma from CRC patients and healthy donors with Ponceau S (Ponc.S) as an internal control. Relative expression was analyzed using ImageJ (version 1.53r) (Healthy donors: n = 8, CRC patients: n = 48 per group; mean ± SEM, Student’s *t*-test). (E) Western blotting analysis of APOA4 expression in plasma from CRC patients and healthy donors. (F) Quantification analysis of APOA4 in plasma from CRC patients and healthy donors with Ponceau S (Ponc.S) as an internal control. Relative expression was analyzed using ImageJ (version 1.53r) (Healthy donors: n = 8, CRC patients: n = 48 per group; mean ± SEM, Student’s *t*-test). Non-significant (ns) *p*>0.05, * *p*<0.05, ** *p*<0.01, *** *p*<0.001, **** *p*<0.0001.

**
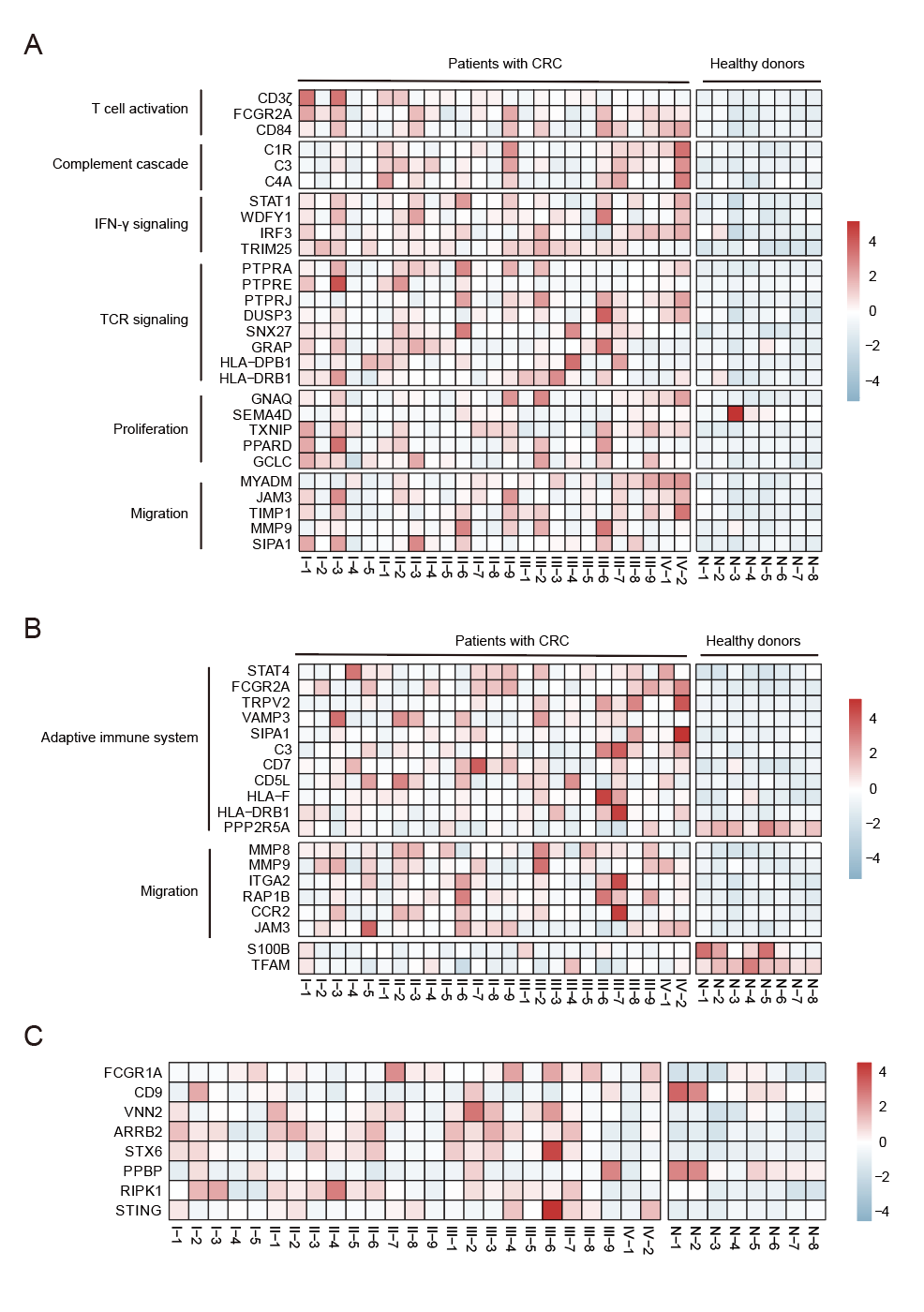
**

**Supplemental Figure 3. Significantly changed proteins in CD4 T cells, CD8 T cells, and monocytes.**

(A) Heatmap of proteins in CD4^+^ T cells that were regulated by the five indicated enriched biological processes. (B) Heatmap of selected proteins in CD8^+^ T cells that regulate the adaptive immune system and migration processes. (C) Heatmap of selected proteins in monocytes.


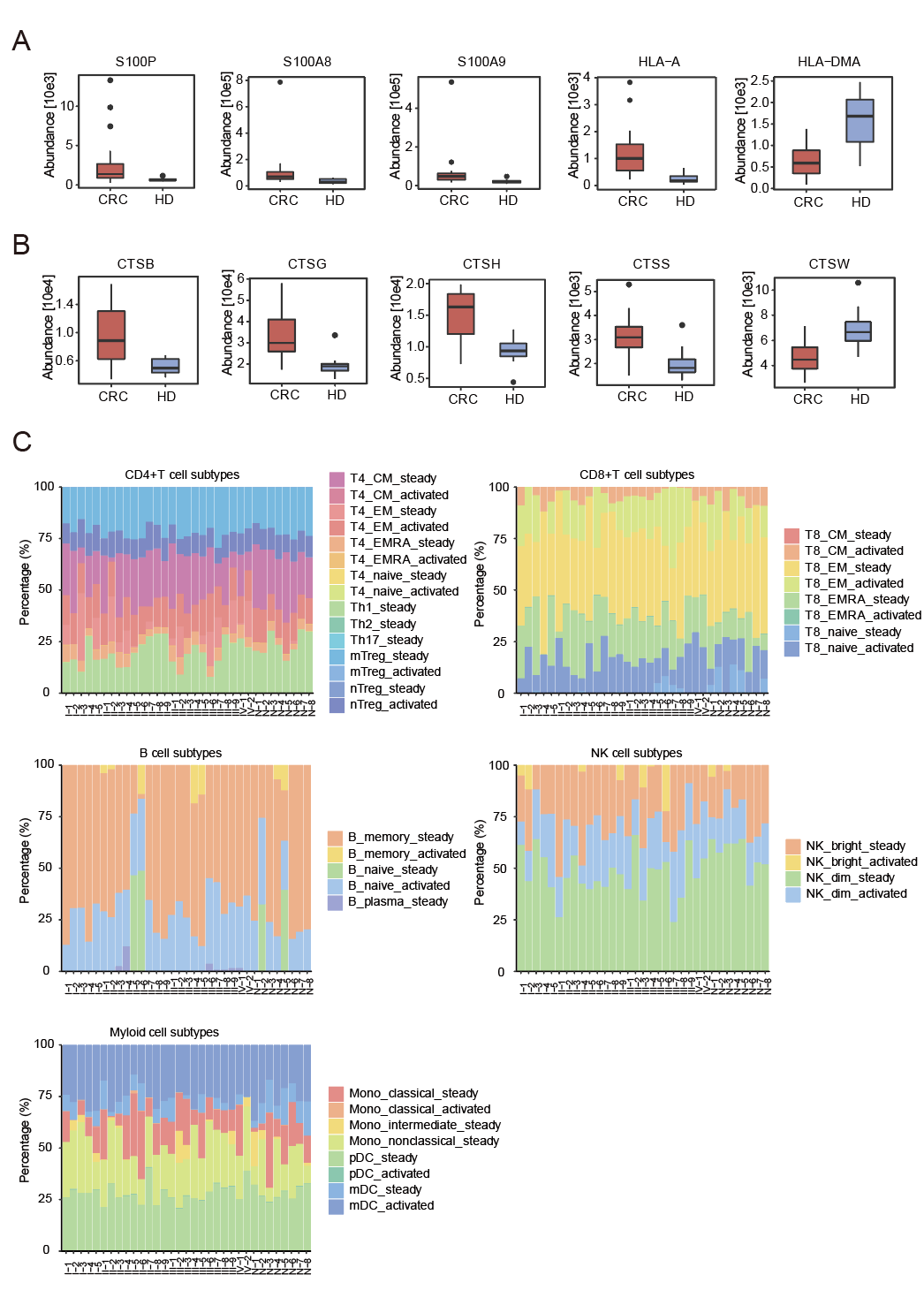


**Supplemental Figure 4. Significantly changed proteins in immune cells and identification of immune cell subtypes.**

(A) Boxplots showing the expression levels of S100P, S100A8, S100A9, HLA-A, and HLA-DMA in B cells from patients with CRC (CRC) and healthy donors (HD). (B) Boxplots showing the expression levels of CTSB, CTSG, CTSH, CTSS, and CTSW in NK cells from patients with CRC (CRC) and healthy donors (HD). (C) The distribution and proportions of cell subsets among the five major immune cell types, as classified by CIBERSORT, are depicted with different colors representing each cell type.


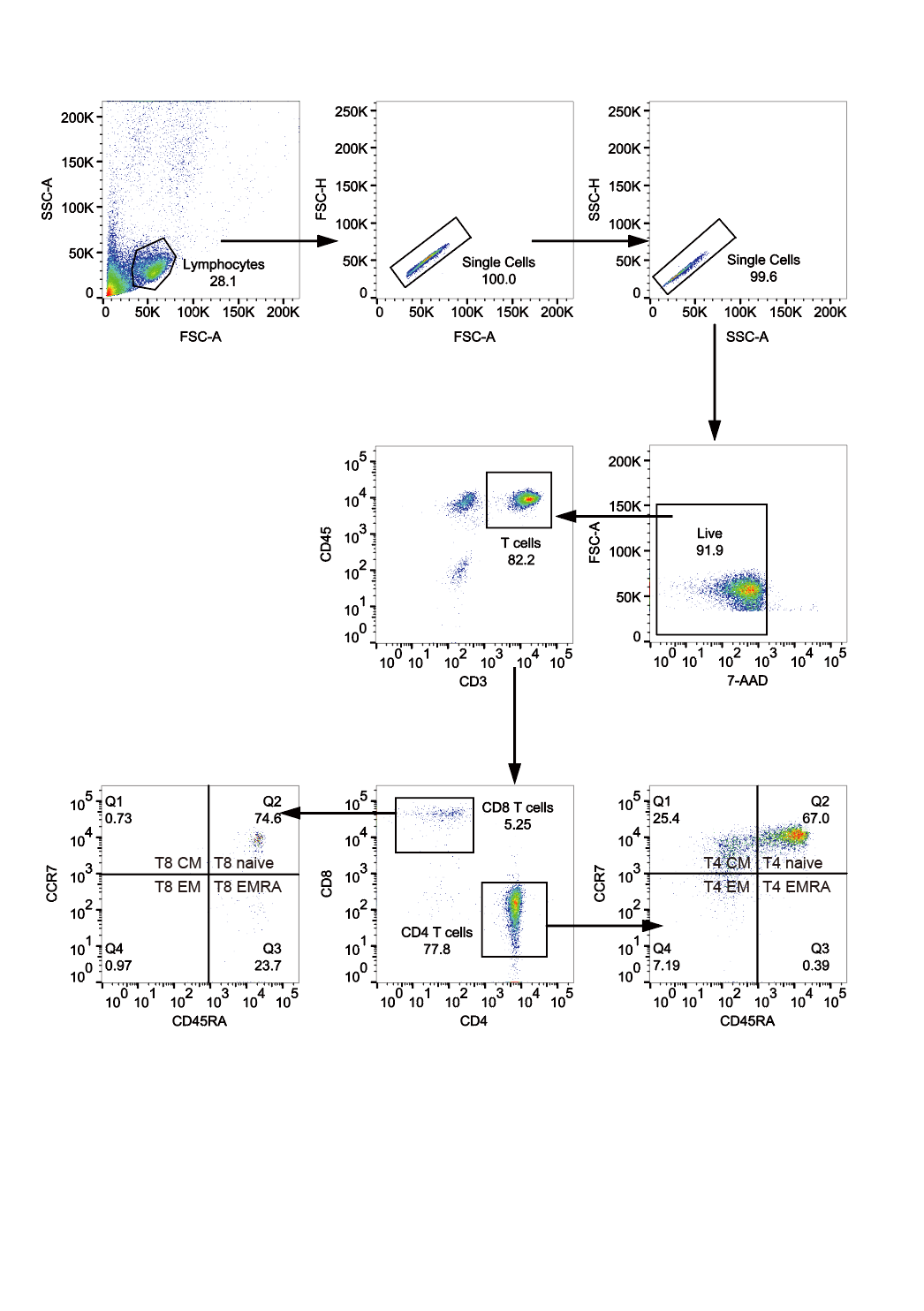


**Supplemental Figure 5. Flow cytometry analysis of peripheral T cell subsets.**

Representative flow cytometry scatter plots illustrate gating strategies for T cell subpopulations in this study.

**
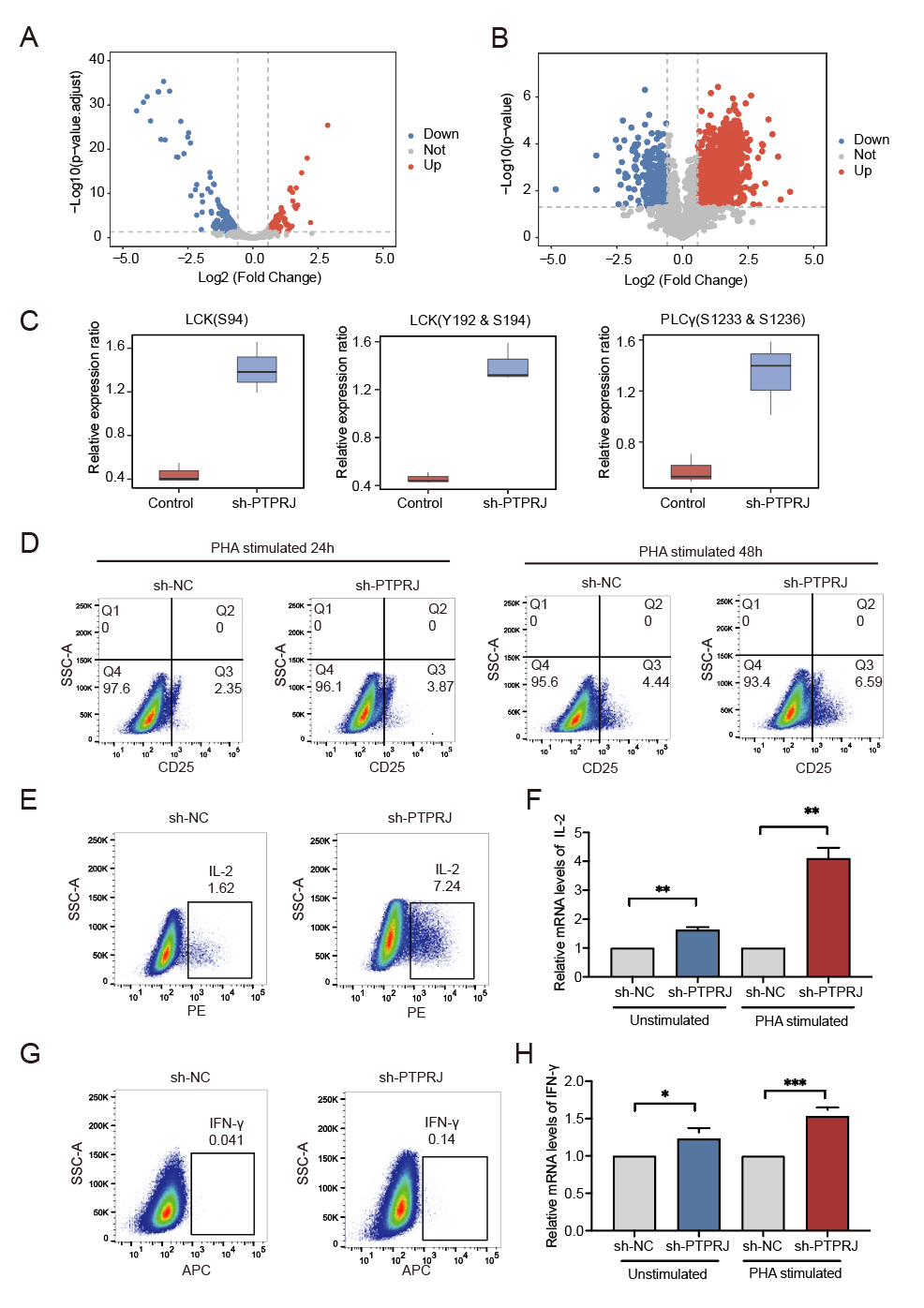
**

**Supplemental Figure 6. PTPRJ regulates T cell activation.**

(A) Proteins that were significantly altered in sh-PTPRJ Jurkat cells, with red representing upregulated and blue representing downregulated proteins. (B) Phosphorylation sites with significant changes in sh-PTPRJ Jurkat cells. Red represents significantly up-regulated phosphorylation sites and blue represents significantly down-regulated phosphorylation sites. (C) Boxplots showing phosphorylation sites associated with the TCR signaling pathway that undergo significant changes in sh-PTPRJ Jurkat cells. (D) Flow cytometry detection of CD25 expression in sh-PTPRJ cells and sh-negative control (NC) cells after stimulation with PHA. (E) Flow cytometry detection of IL-2 expression in sh-PTPRJ cells and sh-NC cells after stimulation with PHA. (F) RT-qPCR was used to detect the mRNA level of IL-2 after stimulation with or without PHA (n = 4 per group; mean ± standard error of the mean, Welch's *t*-test). Non-significant (ns) *p*>0.05, * *p*<0.05, ** *p*<0.01, *** *p*<0.001, **** *p*<0.0001. (G) Flow cytometry detection of IFN-γ expression in sh-PTPRJ cells and sh-NC cells after stimulation with PHA. (H) RT-qPCR was used to detect the mRNA level of IFN-γ after stimulation with or without PHA (n = 4 per group; mean ± standard error of the mean, Welch's *t*-test). Non-significant (ns) *p*>0.05, * *p*<0.05, ** *p*<0.01, *** *p*<0.001, **** *p*<0.0001.

**Supplemental Tables**

**Supplemental Table S1. Pathological characteristics of patients with CRC included in the plasma proteome analysis**

| Information | | Num (%) | Information | | Num (%) |
| --- | --- | --- | --- | --- | --- |
| Sex | Male | 29 (71%) | Stage | I | 12 (29%) |
|  | Female | 12 (29%) |  | II | 12 (29%) |
| Age (years) | > 60 | 29 (71%) |  | III | 12 (29%) |
|  | < 60 | 12 (29%) |  | IV | 5 (13%) |
| Site* | Right | 9 (22%) | T | 1 | 3 (7%) |
|  | Left | 32 (78%) |  | 2 | 10 (25%) |
| Differentiation degree | High | 2 (5%) |  | 3 | 14 (34%) |
|  | Medium | 39 (95%) |  | 4 | 14 (34%) |
| Nerve infiltration | 0 | 33 (80%) | N | 0 | 29 (71%) |
|  | 1 | 8 (20%) |  | 1 | 12 (29%) |
| Vascular infiltration | 0 | 30 (73%) | M | 0 | 36 (88%) |
|  | 1 | 11 (27%) |  | 1 | 5 (12%) |

Site*: Right: cecum, ascending colon, transverse colon, hepatic flexure of the colon; Left: descending colon, rectosigmoid, rectum, sigmoid colon.

**Supplemental Table S2. Pathological characteristics of patients with CRC included in the five immune cell proteome analysis**

| Information | | Num (%) | Information | | Num (%) |
| --- | --- | --- | --- | --- | --- |
| Sex | Male | 20 (80%) | Stage | I | 5 (20%) |
|  | Female | 5 (20%) |  | II | 9 (36%) |
| Age (years) | > 60 | 17 (68%) |  | III | 9 (36%) |
|  | < 60 | 8 (32%) |  | IV | 2 (8%) |
| Site* | Right | 3 (12%) | T | 1 | 2 (8%) |
|  | Left | 22 (88%) |  | 2 | 4 (16%) |
| Differentiation degree | High | 1 (4%) |  | 3 | 10 (40%) |
|  | Medium | 24 (96%) |  | 4 | 9 (36%) |
| Nerve infiltration | 0 | 19 (76%) | N | 0 | 16 (64%) |
|  | 1 | 6 (24%) |  | 1 | 9 (36%) |
| Vascular infiltration | 0 | 17 (92%) | M | 0 | 23 (92%) |
|  | 1 | 8 (8%) |  | 1 | 2 (8%) |

Site*: Right: cecum, ascending colon, transverse colon, hepatic flexure of the colon; Left: descending colon, rectosigmoid, rectum, sigmoid colon.

**Supplemental Table S3. Microbeads and antibodies used in this study**

| Name | Source | Catalog number |
| --- | --- | --- |
| CD56 MicroBeads, human | Miltenyi Biotec | 130-050-401 |
| CD8 MicroBeads, human | Miltenyi Biotec | 130-045-201 |
| CD4 MicroBeads, human | Miltenyi Biotec | 130-045-101 |
| CD19 MicroBeads, human | Miltenyi Biotec | 130-050-301 |
| CD14 MicroBeads, human | Miltenyi Biotec | 130-050-201 |
| BD Pharmingen™ PE Mouse Anti-Human CD56 | BD | 561903 |
| BD Pharmingen™ FITC Mouse Anti-Human CD16 | BD | 555406 |
| BD Pharmingen™ PE Mouse Anti-Human CD8 | BD | 560959 |
| BD Pharmingen™ FITC Mouse Anti-Human CD3 | BD | 555339 |
| BD Pharmingen™ PE Mouse Anti-Human CD4 | BD | 561843 |
| BD Pharmingen™ PE Mouse Anti-Human CD19 | BD | 561741 |
| BD Pharmingen™ PE Mouse Anti-Human CD14 | BD | 561707 |
| BD Pharmingen™ FITC Mouse Anti-Human CD45 | BD | 560976 |
| PE Anti-Human IL-2 Antibody | BioLegend | 500306 |
| APC Anti-Human IFN-γ Antibody | BioLegend | 502511 |
| PE Anti-Human CD25 Antibody | BioLegend | 302605 |
| BD Horizon™ V500 Mouse Anti-Human CD45 | BD | 560779 |
| Pacific Blue Anti-Human CD3 Antibody | BioLegend | 317313 |
| APC/Cy7 Anti-Human CD4 Antibody | BioLegend | 300517 |
| FITC Anti-Human CD8 Antibody | BioLegend | 300905 |
| APC Anti-Human CD45RA Antibody | BioLegend | 304111 |
| PE Anti-Human CCR7 Antibody | BioLegend | 353203 |
| PTPRJ Polyclonal Antibody | proteintech | 55123-1-AP |
| Anti-LRG1/LRG Antibody [EPR12362] | abcam | ab178698 |
| APOA4 Polyclonal Antibody | proteintech | 17996-1-AP |
| α-Tubulin Polyclonal Antibody | Beyotime | AF5012 |

**Supplemental Table S4. shRNA and qPCR primers used in the present study**

| Primer | Sequence (5' to 3') |
| --- | --- |
| sh-NC-F | CCGGTTCTCCGAACGTGTCACGTTTCTCGAGAAACGTGACACGTTCGGAGAATTTTTTCTAGAG |
| sh-NC-R | AATTCTCTAGAAAAAATTCTCCGAACGTGTCACGTTTCTCGAGAAACGTGACACGTTCGGAGAA |
| sh-PTPRJ-1 | CCGGCCACACAAGCACGTATGACAACTCGAGTTGTCATACGTGCTTGTGTGGTTTTT |
| sh-PTPRJ-2 | CCGGGCCATAGAGTTCAGGACAAATCTCGAGATTTGTCCTGAACTCTATGGCTTTTT |
| sh-PTPRJ-3 | CCGGCCGATACAATGCCACCGTTTACTCGAGTAAACGGTGGCATTGTATCGGTTTTT |
| sh-PTPRJ-4 | CCGGCCTACTGTGTCTTGGAATCTACTCGAGTAGATTCCAAGACACAGTAGGTTTTT |
| GAPDH-F | ACAACTTTGGTATCGTGGAAGG |
| GAPDH-R | GCCATCACGCCACAGTTTC |
| PTPRJ-F | GGCACCCCTAGTCCAATTCC |
| PTPRJ-R | TCCCATTAGATCCTTGTTCAGGT |
| IL2-F | AGAACTCAAACCTCTGGAGGAAG |
| IL2-R | GCTGTCTCATCAGCATATTCACAC |
| IFNγ-F | TCGGTAACTGACTTGAATGTCCA |
| IFNγ-R | TCGCTTCCCTGTTTTAGCTGC |
