# Supplementary material for: Quantitative Proteomic Analysis Reveals Functional Alterations of the Peripheral Immune System in Colorectal Cancer
Source: Mol Cell Proteomics. 2024 May 11;23(6):100784. doi: 10.1016/j.mcpro.2024.100784 (PMC11215959; doi:10.1016/j.mcpro.2024.100784)

**Annotated spectra for proteins with single unique peptide**

| Dataset | Accession | Peptide | Spectra number |
| --- | --- | --- | --- |
| Plasma | G3V2B9 [1-7] | [-R].MLSLGTK.[A] | 1 |

| Dataset | Accession | Peptide | Spectra number |
| --- | --- | --- | --- |
| Plamsa | P0CI25 [205-211] | [K].GKEIFHR.[L] | 1 |

| Dataset | Accession | Peptide | Spectra number |
| --- | --- | --- | --- |
| CD4T | A0A0G2JPR0  [1100-1126] | [K].LQETSNWLLSQQQADGSF  QDPCPVLDR.[S] | 1 |

| Dataset | Accession | Peptide | Spectra number |
| --- | --- | --- | --- |
| CD4T | A0A2R8Y793 [300-309] | [K].EITALAPSTM.[-] | 1 |

| Dataset | Accession | Peptide | Spectra number |
| --- | --- | --- | --- |
| CD4T | F5GZG1 [1-17] | [-].MLEILDTAGTEQFTAMR.[D] | 3 |

| Dataset | Accession | Peptide | Spectra number |
| --- | --- | --- | --- |
| CD4T | J3KPM9 [706-714] | [K].TELISVSEV.[-] | 1 |

| Dataset | Accession | Peptide | Spectra number |
| --- | --- | --- | --- |
| CD4T | P11217 [657-681] | [K].VIPAADLSEQISTAGTEASGTGNMK.[F] | 2 |

| Dataset | Accession | Peptide | Spectra number |
| --- | --- | --- | --- |
| CD4T | P47897 [35-50] | [R].EAATQAQQTLGSTIDK.[A] | 1 |

| Dataset | Accession | Peptide | Spectra number |
| --- | --- | --- | --- |
| CD4T | P47897 [35-50] | [R].EAATQAQQTLGSTIDK.[A] | 1 |

| Dataset | Accession | Peptide | Spectra number |
| --- | --- | --- | --- |
| CD4T | Q03181 [259-265] | [R].ELTEFAK.[S] | 1 |

| Dataset | Accession | Peptide | Spectra number |
| --- | --- | --- | --- |
| CD4T | Q13303-2 [17-26] | [R].QTGSPGMIYR.[N] | 2 |

| Dataset | Accession | Peptide | Spectra number |
| --- | --- | --- | --- |
| CD4T | Q15697 [116-126] | [K].EIVTLVEDFHR.[A] | 1 |

| Dataset | Accession | Peptide | Spectra number |
| --- | --- | --- | --- |
| CD4T | Q15836 [15-30] | [R].LQQTQNQVDEVVDIMR.[V] | 1 |

| Dataset | Accession | Peptide | Spectra number |
| --- | --- | --- | --- |
| CD4T | Q8TDG2 [77-87] | [R].GLVTGWDDMEK.[V] | 2 |

| Dataset | Accession | Peptide | Spectra number |
| --- | --- | --- | --- |
| CD4T | Q93070 [139-145] | [R].AMASVAR.[T] | 1 |

| Dataset | Accession | Peptide | Spectra number |
| --- | --- | --- | --- |
| CD8T | C9JBU3 [1-15] | [-].MSPVDDTFISGSLDK.[T] | 1 |

| Dataset | Accession | Peptide | Spectra number |
| --- | --- | --- | --- |
| CD8T | E7ESP4 [534-553] | [-].EGILGQHQFLEGPEGIENTR.[F] | 1 |

| Dataset | Accession | Peptide | Spectra number |
| --- | --- | --- | --- |
| CD8T | F5GZG1 [1-17] | [-].MLEILDTAGTEQFTAMR.[D] | 2 |

| Dataset | Accession | Peptide | Spectra number |
| --- | --- | --- | --- |
| CD8T | I3L4N8 [230-241] | [K].SYELPDGQVITI.[-] | 1 |

| Dataset | Accession | Peptide | Spectra number |
| --- | --- | --- | --- |
| CD8T | K7EM38 [124-133] | [R].TTGIVMDSGD.[-] | 2 |

| Dataset | Accession | Peptide | Spectra number |
| --- | --- | --- | --- |
| CD8T | O43866 [257-262] | [R].LEVLHK.[G] | 1 |

| Dataset | Accession | Peptide | Spectra number |
| --- | --- | --- | --- |
| CD8T | P12814 [773-794] | [K].ACLISLGYDIGNDPQGEAEFAR.[I] | 1 |

| Dataset | Accession | Peptide | Spectra number |
| --- | --- | --- | --- |
| CD8T | P24158 [92-115] | [R].TQEPTQQHFSVAQVFLNNYDAENK.[L] | 1 |

| Dataset | Accession | Peptide | Spectra number |
| --- | --- | --- | --- |
| CD8T | P47897 [35-50] | [R].EAATQAQQTLGSTIDK.[A] | 1 |

| Dataset | Accession | Peptide | Spectra number |
| --- | --- | --- | --- |
| CD8T | P59665 [63-69] | [K].NMACYCR.[I] | 1 |

| Dataset | Accession | Peptide | Spectra number |
| --- | --- | --- | --- |
| CD8T | Q03181 [259-265] | [R].ELTEFAK.[S] | 1 |

| Dataset | Accession | Peptide | Spectra number |
| --- | --- | --- | --- |
| CD8T | Q15836 [15-30] | [R].LQQTQNQVDEVVDIMR.[V] | 2 |

| Dataset | Accession | Peptide | Spectra number |
| --- | --- | --- | --- |
| CD8T | Q8N257 [94-100] | [R].EVQTAVR.[L] | 1 |

| Dataset | Accession | Peptide | Spectra number |
| --- | --- | --- | --- |
| CD8T | Q8TDG2 [77-87] | [R].GLVTGWDDMEK.[L] | 2 |

| Dataset | Accession | Peptide | Spectra number |
| --- | --- | --- | --- |
| CD8T | Q93070 [139-145] | [R].AMASVAR.[T] | 1 |

| Dataset | Accession | Peptide | Spectra number |
| --- | --- | --- | --- |
| CD8T | Q9NZJ4 [4300-4308] | [K].VNSLPEILK.[E] | 1 |

| Dataset | Accession | Peptide | Spectra number |
| --- | --- | --- | --- |
| Monocyte | P09493-3 [38-48] | [K].QLEDELVSLQK.[K] | 1 |

| Dataset | Accession | Peptide | Spectra number |
| --- | --- | --- | --- |
| Monocyte | A0A2R8YGF8  [148-156] | [R].TTGIVMDSGDGVTHTVPI.[-] | 2 |

| Dataset | Accession | Peptide | Spectra number |
| --- | --- | --- | --- |
| Monocyte | E7EX18 [148-156] | [R].GLQEHQR.[G] | 1 |

| Dataset | Accession | Peptide | Spectra number |
| --- | --- | --- | --- |
| Monocyte | Q8TDG2 [77-87] | [R].GLVTGWDDMEK.[L] | 2 |

| Dataset | Accession | Peptide | Spectra number |
| --- | --- | --- | --- |
| B | Q03181 [259-265] | [R].ELTEFAK.[S] | 1 |

| Dataset | Accession | Peptide | Spectra number |
| --- | --- | --- | --- |
| B | Q8TDG2 [77-87] | [R].GLVTGWDDMEK.[L] | 1 |

| Dataset | Accession | Peptide | Spectra number |
| --- | --- | --- | --- |
| NK | A0A2R8Y793 [300-309] | [K].EITALAPSTM.[-] | 1 |

| Dataset | Accession | Peptide | Spectra number |
| --- | --- | --- | --- |
| NK | A0A2R8YGF8 [148-165] | [R].TTGIVMDSGDGVTHTVPI.[-] | 1 |

| Dataset | Accession | Peptide | Spectra number |
| --- | --- | --- | --- |
| NK | D6R9G1 [1-10] | [-].METDLEVIIK.[D] | 1 |

| Dataset | Accession | Peptide | Spectra number |
| --- | --- | --- | --- |
| NK | E9PK25 [191-204] | [K].LGGSAVISLEGKPL.[-] | 1 |

| Dataset | Accession | Peptide | Spectra number |
| --- | --- | --- | --- |
| NK | O43866 [257-262] | [R].LEVLHK.[G] | 1 |

| Dataset | Accession | Peptide | Spectra number |
| --- | --- | --- | --- |
| NK | Q03181 [259-265] | [R].ELTEFAK.[S] | 1 |

| Dataset | Accession | Peptide | Spectra number |
| --- | --- | --- | --- |
| NK | Q06055-2 [124-130] | [R].DIDTAAK.[F] | 1 |

| Dataset | Accession | Peptide | Spectra number |
| --- | --- | --- | --- |
| NK | Q13303-2 [17-26] | [R].QTGSPGMIYR.[N] | 1 |

| Dataset | Accession | Peptide | Spectra number |
| --- | --- | --- | --- |
| NK | Q15836 [15-30] | [R].LQQTQNQVDEVVDIMR.[V] | 1 |

| Dataset | Accession | Peptide | Spectra number |
| --- | --- | --- | --- |
| NK | Q5R341 [321-342] | [K].AISCEPLESPVHGSMDCDPDLR.[A] | 1 |

| Dataset | Accession | Peptide | Spectra number |
| --- | --- | --- | --- |
| NK | Q7Z2K8 [606-613] | [K].VGSLPLEK.[G] | 1 |

| Dataset | Accession | Peptide | Spectra number |
| --- | --- | --- | --- |
| NK | Q8TDG2 [77-87] | [R].GLVTGWDDMEK.[L] | 1 |

| Dataset | Accession | Peptide | Spectra number |
| --- | --- | --- | --- |
| NK | Q9ULE3 [754-766] | [R].HLVCVFASLLLER.[R] | 1 |

| Dataset | Accession | Peptide | Spectra number |
| --- | --- | --- | --- |
| sh-PTPRJ | Q9UKV8 [385-395] | [R]. SASFNTDPYVR.[E] | 1 |


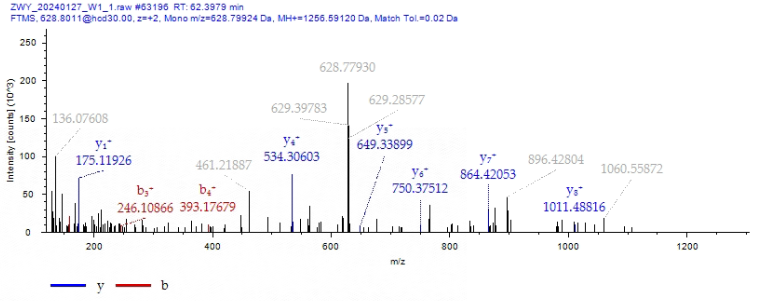


| Dataset | Accession | Peptide | Spectra number |
| --- | --- | --- | --- |
| sh-PTPRJ | Q9H9G7 [413-424] | [R].VLPAPMLQYGGR.[N] | 1 |


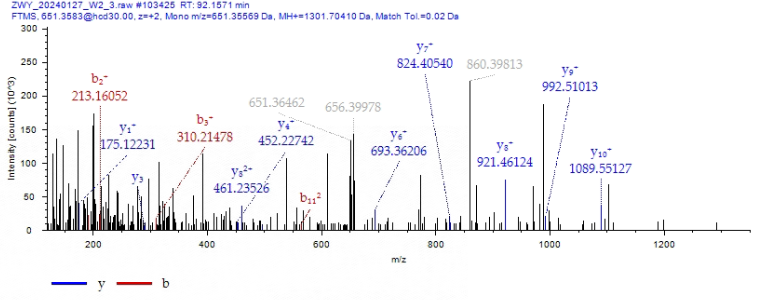


| Dataset | Accession | Peptide | Spectra number |
| --- | --- | --- | --- |
| sh-PTPRJ | Q9Y672 [89-101] | [K].FINPDWIALHTSR.[G] | 1 |


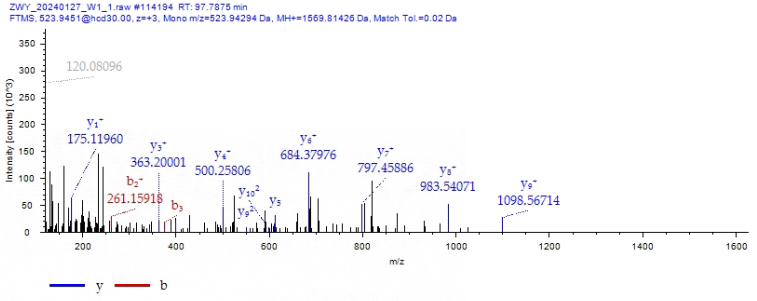


| Dataset | Accession | Peptide | Spectra number |
| --- | --- | --- | --- |
| sh-PTPRJ | O95817 [194-203] | [R].SSLGSHQLPR.[G] | 1 |


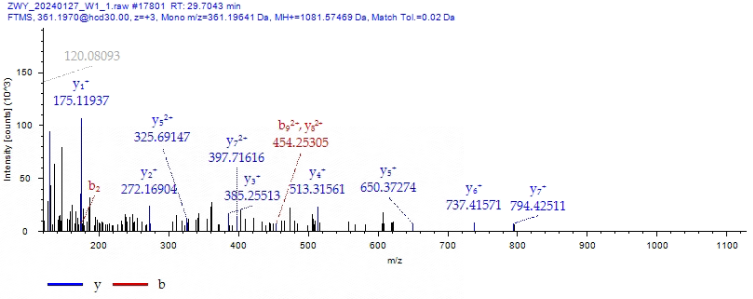


| Dataset | Accession | Peptide | Spectra number |
| --- | --- | --- | --- |
| sh-PTPRJ | Q9H8M2 [229-235] | [K].ILHAGFK.[M] | 1 |


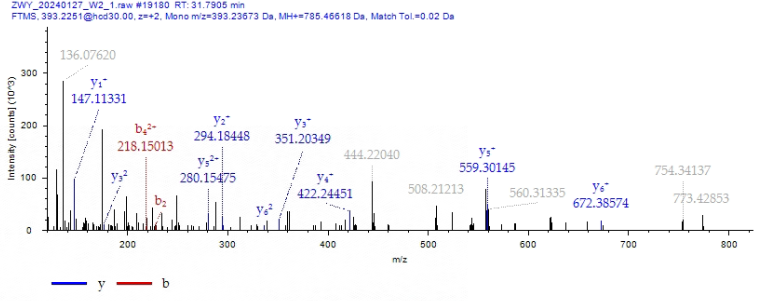


| Dataset | Accession | Peptide | Spectra number |
| --- | --- | --- | --- |
| sh-PTPRJ | O75794 [25-35] | [K].SVILPLPQNVK.[D] | 1 |


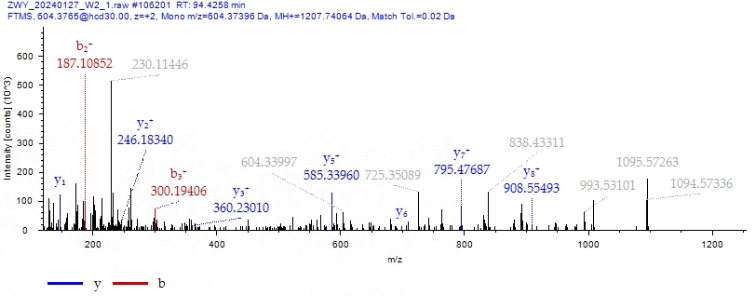


| Dataset | Accession | Peptide | Spectra number |
| --- | --- | --- | --- |
| sh-PTPRJ | Q9P003 [85-104] | [R].YIMVPSGNMGVFDPTEIHNR.[G] | 1 |


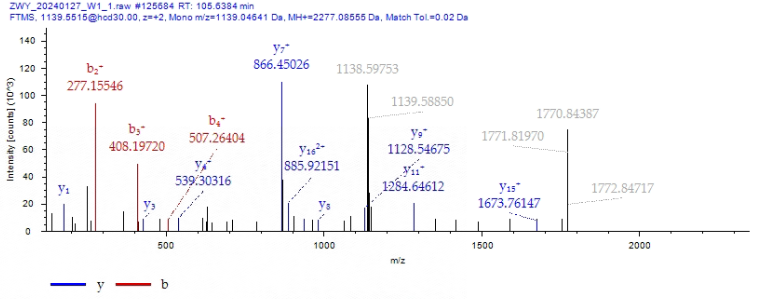


| Dataset | Accession | Peptide | Spectra number |
| --- | --- | --- | --- |
| sh-PTPRJ | P12074 [61-78] | [K].SHHGEHERPEFIAYPHLR.[I] | 1 |


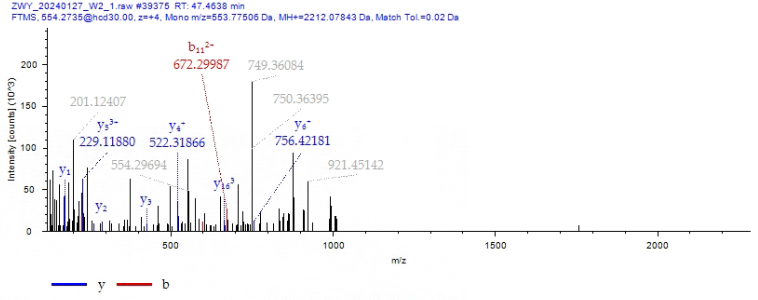


| Dataset | Accession | Peptide | Spectra number |
| --- | --- | --- | --- |
| sh-PTPRJ | Q5TZA2 [1676-1685] | [R].EAQAQALQDR.[V] | 1 |


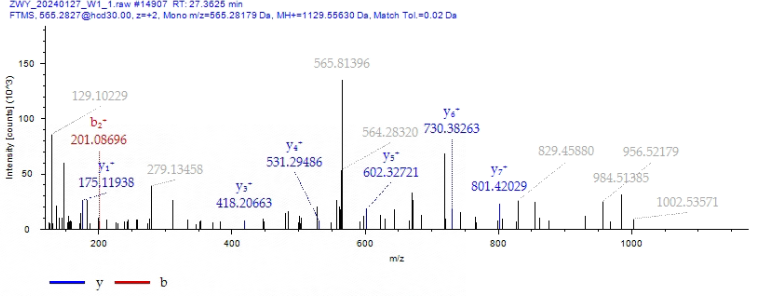


| Dataset | Accession | Peptide | Spectra number |
| --- | --- | --- | --- |
| sh-PTPRJ | H7BZ55 [553-559] | [R].LQQLEEK.[V] | 1 |


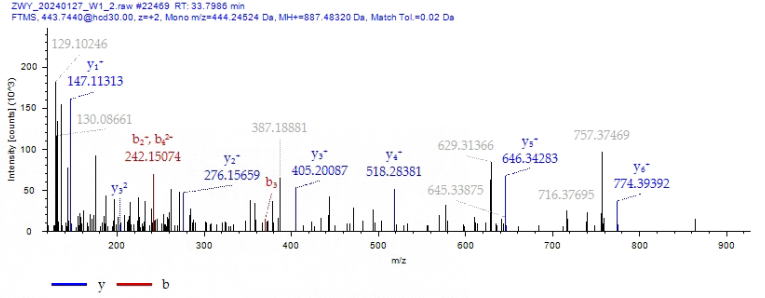


| Dataset | Accession | Peptide | Spectra number |
| --- | --- | --- | --- |
| sh-PTPRJ | P35221 [106-120] | [K].AAAGEFADDPCSSVK.[R] | 1 |


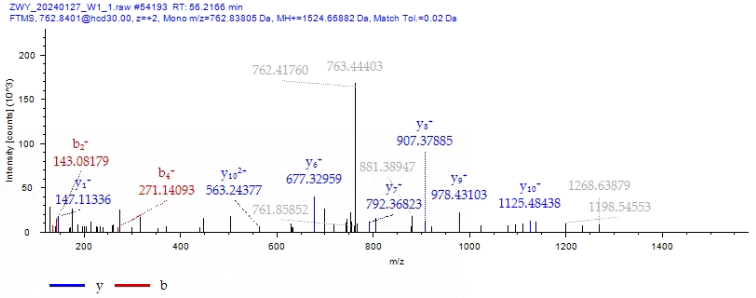


| Dataset | Accession | Peptide | Spectra number |
| --- | --- | --- | --- |
| sh-PTPRJ | Q8NBI2 [179-184] | [K].LFFSLK.[N] | 1 |


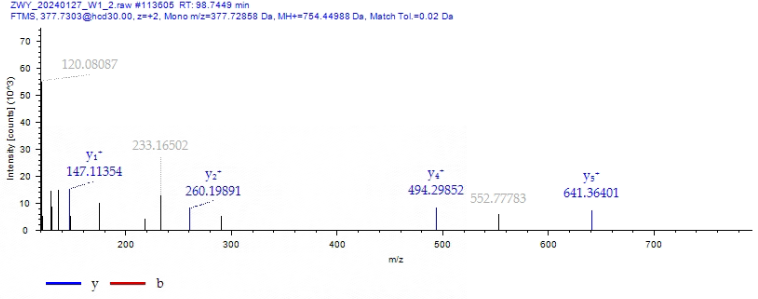


| Dataset | Accession | Peptide | Spectra number |
| --- | --- | --- | --- |
| sh-PTPRJ | Q9H147 [265-275] | [K].MAYLLIEEDIR.[D] | 1 |


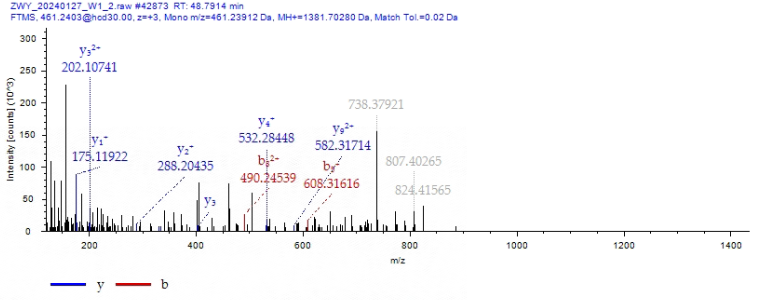


| Dataset | Accession | Peptide | Spectra number |
| --- | --- | --- | --- |
| sh-PTPRJ | Q8NBA8 [204-213] | [K].TSISSQYVIR.[M] | 1 |


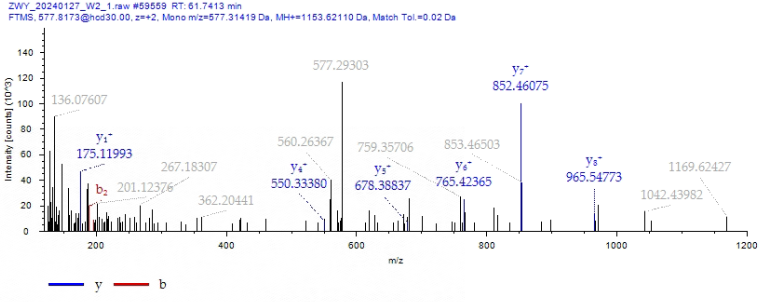


| Dataset | Accession | Peptide | Spectra number |
| --- | --- | --- | --- |
| sh-PTPRJ | Q96FJ2 [10-31] | [K].NADMSEDMQQDAVDCATQAMEK.[Y] | 1 |


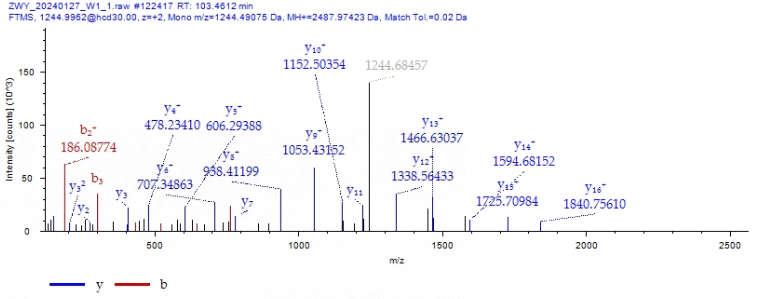


| Dataset | Accession | Peptide | Spectra number |
| --- | --- | --- | --- |
| sh-PTPRJ | Q8IUD2 [1038-1043] | [R].DPLILR.[G] | 1 |


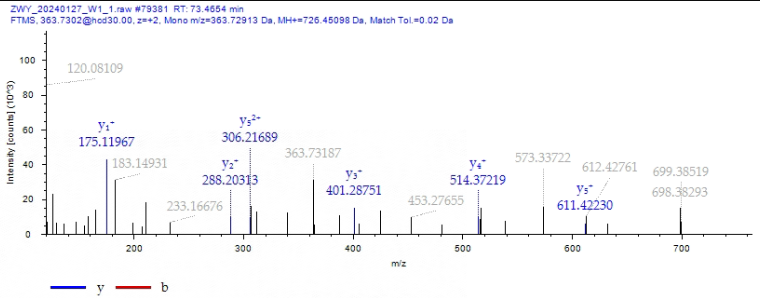


| Dataset | Accession | Peptide | Spectra number |
| --- | --- | --- | --- |
| sh-PTPRJ | Q9H6T0 [205-215] | [K].TMVAVILHLLK.[E] | 1 |


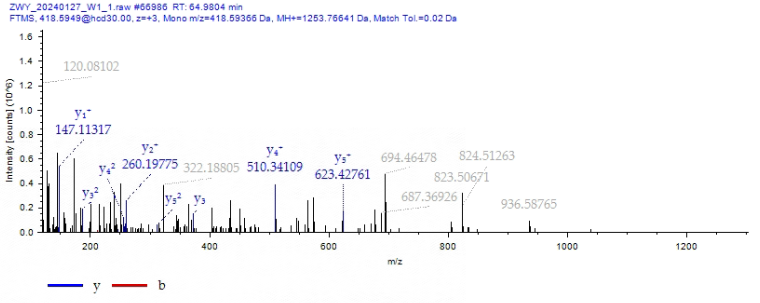


| Dataset | Accession | Peptide | Spectra number |
| --- | --- | --- | --- |
| sh-PTPRJ | Q9NXK8 [180-188] | [R].SLVLGGTYR.[V] | 1 |


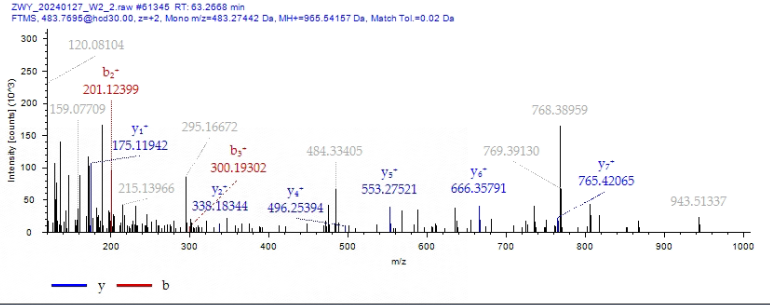


| Dataset | Accession | Peptide | Spectra number |
| --- | --- | --- | --- |
| sh-PTPRJ | P17900 [170-179] | [R]. IESVLSSSGK.[R] | 1 |


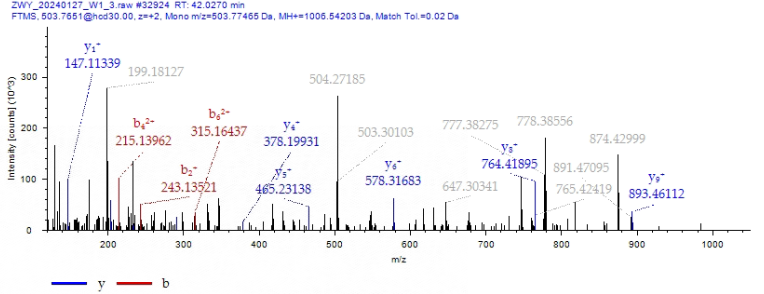


| Dataset | Accession | Peptide | Spectra number |
| --- | --- | --- | --- |
| sh-PTPRJ | O15379 [61-72] | [R].FHSEDYIDFLQR.[V] | 1 |


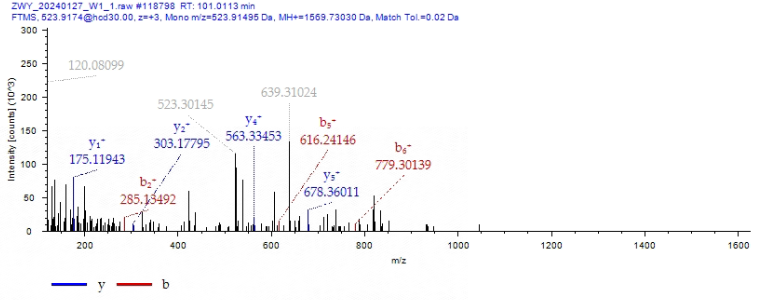


| Dataset | Accession | Peptide | Spectra number |
| --- | --- | --- | --- |
| sh-PTPRJ | Q9Y3E1 [29-43] | [R].IDELPEGAVKPPANK.[Y] | 1 |


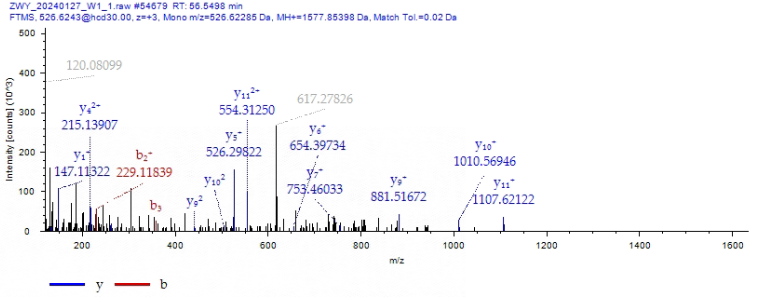


| Dataset | Accession | Peptide | Spectra number |
| --- | --- | --- | --- |
| sh-PTPRJ | P14210 [171-178] | [K].DLQENYCR.[N] | 1 |


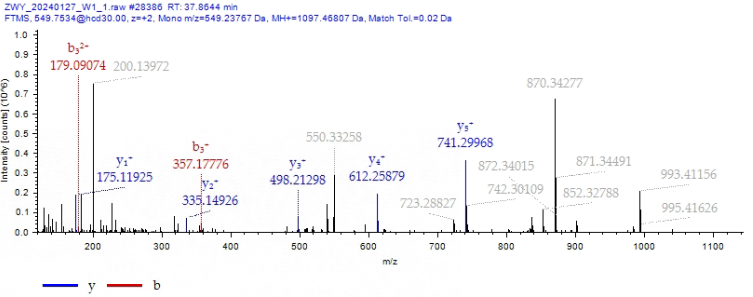


| Dataset | Accession | Peptide | Spectra number |
| --- | --- | --- | --- |
| sh-PTPRJ | A8MV81 [55-63] | [K].MSIHLIHMR.[V] | 1 |


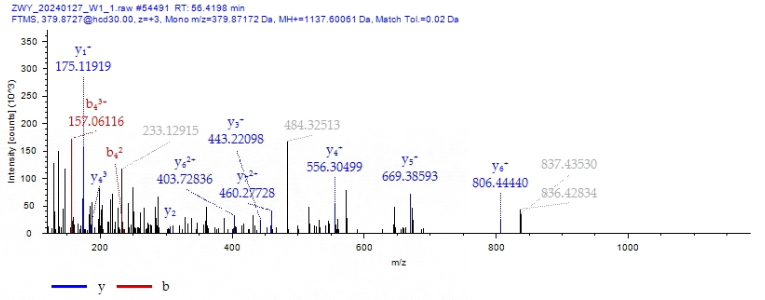


| Dataset | Accession | Peptide | Spectra number |
| --- | --- | --- | --- |
| sh-PTPRJ | P01112 [89-97] | [K].SFEDIHQYR.[E] | 1 |


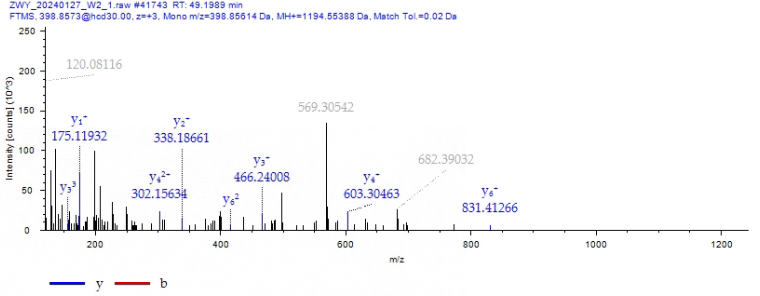


| Dataset | Accession | Peptide | Spectra number |
| --- | --- | --- | --- |
| sh-PTPRJ | Q9UL03 [168-174] | [R].LFALVLR.[L] | 1 |


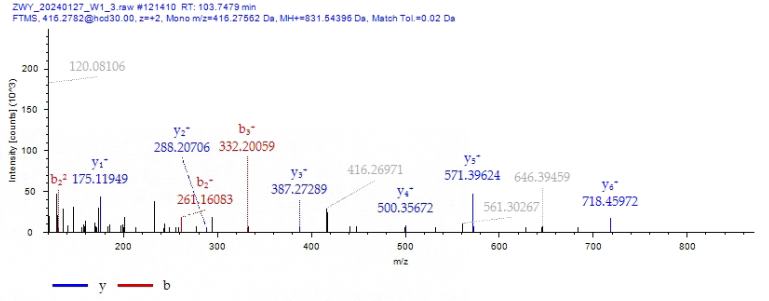


| Dataset | Accession | Peptide | Spectra number |
| --- | --- | --- | --- |
| sh-PTPRJ | Q9HCM3 [1517-1523] | [K].EIQTALR.[H] | 1 |


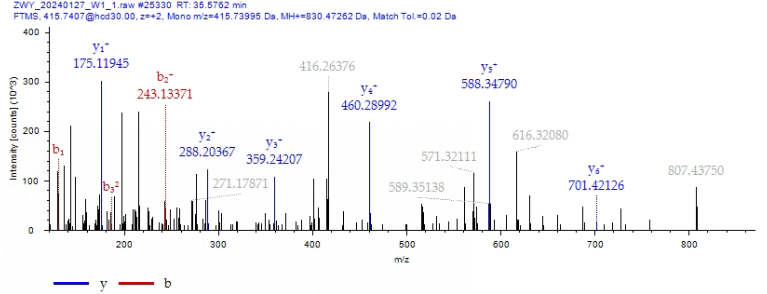


| Dataset | Accession | Peptide | Spectra number |
| --- | --- | --- | --- |
| sh-PTPRJ | P07942 [1377-1384] | [R].LLDELAGK.[L] | 1 |


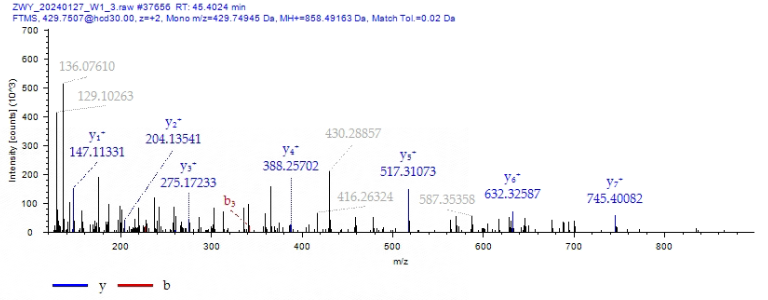


| Dataset | Accession | Peptide | Spectra number |
| --- | --- | --- | --- |
| sh-PTPRJ | Q05C16 [369-374] | [K].NLQILK.[DL] | 1 |


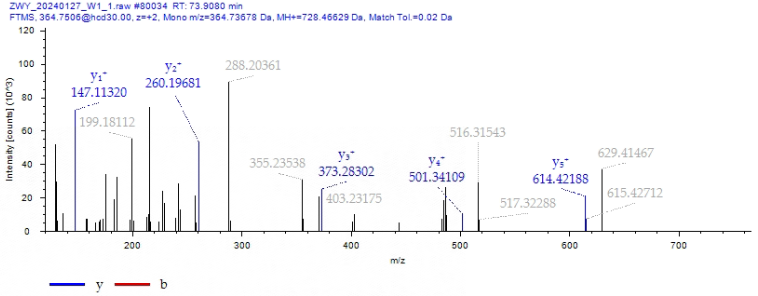


| Dataset | Accession | Peptide | Spectra number |
| --- | --- | --- | --- |
| sh-PTPRJ | P46734 [236-243] | [R].INPELNQK.[G] | 1 |


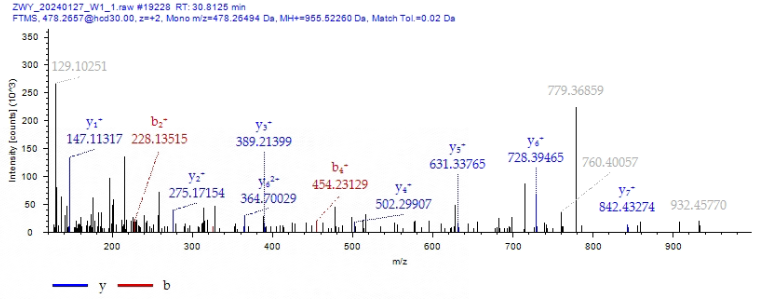


| Dataset | Accession | Peptide | Spectra number |
| --- | --- | --- | --- |
| sh-PTPRJ | Q6P9B6 [129-144] | [K].FTEDLVGSVVHVLSHR.[Q] | 1 |


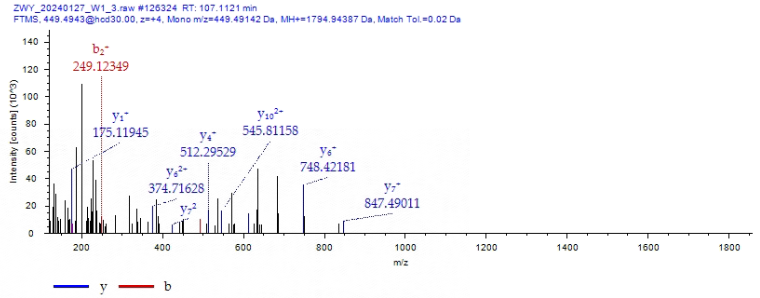


| Dataset | Accession | Peptide | Spectra number |
| --- | --- | --- | --- |
| sh-PTPRJ | Q9H8H3 [94-105] | [R].VTCIDPNPNFEK.[F] | 1 |


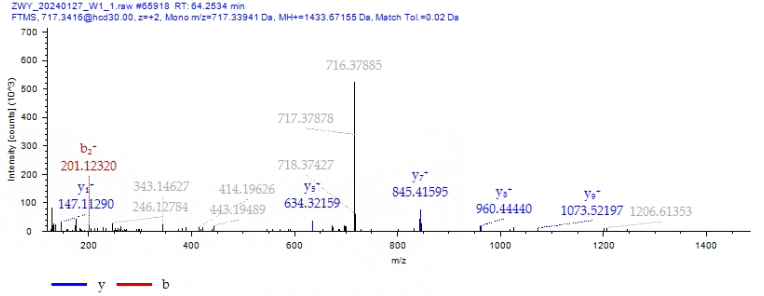


| Dataset | Accession | Peptide | Spectra number |
| --- | --- | --- | --- |
| sh-PTPRJ | Q13015 [48-59] | [K].MIGQATAADQEK.[N] | 1 |


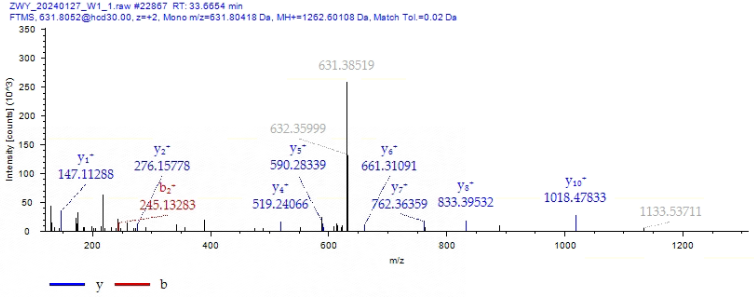


| Dataset | Accession | Peptide | Spectra number |
| --- | --- | --- | --- |
| sh-PTPRJ | Q7L1V2 [259-266] | [R].DALGALLR.[R] | 1 |


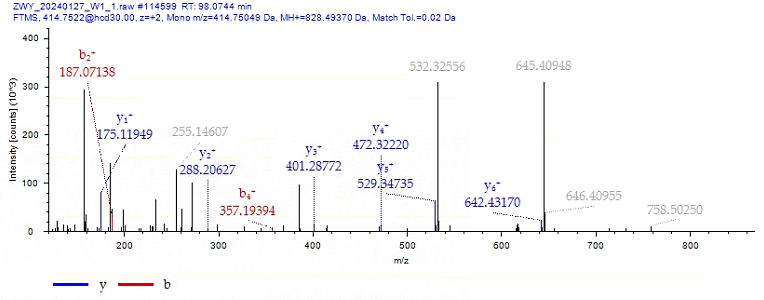


| Dataset | Accession | Peptide | Spectra number |
| --- | --- | --- | --- |
| sh-PTPRJ | Q7Z3U7 [861-870] | [R].LQLLLLNPLK.[E] | 1 |


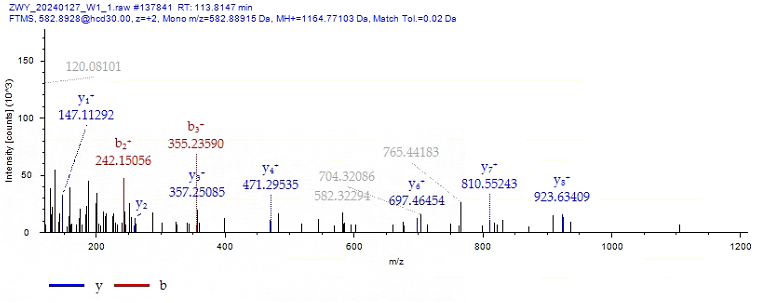


| Dataset | Accession | Peptide | Spectra number |
| --- | --- | --- | --- |
| sh-PTPRJ | O75352 [45-58] | [K].GLGLGIVAGSLLVK.[L] | 1 |


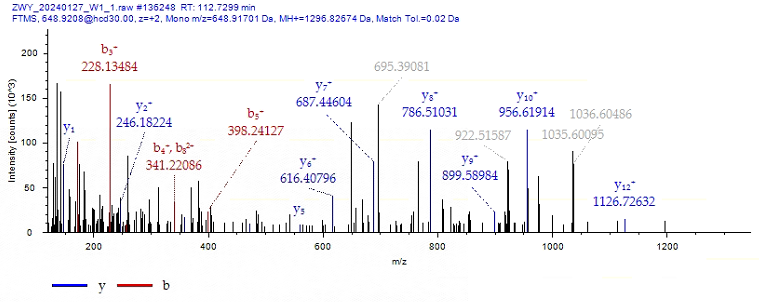


| Dataset | Accession | Peptide | Spectra number |
| --- | --- | --- | --- |
| sh-PTPRJ | Q7Z7H8 [170-188] | [R].TVPFLPLLGGCIDDTILSR.[Q] | 1 |


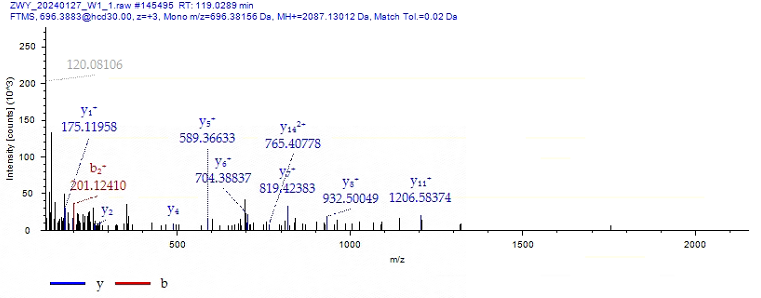


| Dataset | Accession | Peptide | Spectra number |
| --- | --- | --- | --- |
| sh-PTPRJ | Q14197 [103-116] | [R].FHLATAEWIAEPVR.[Q] | 1 |


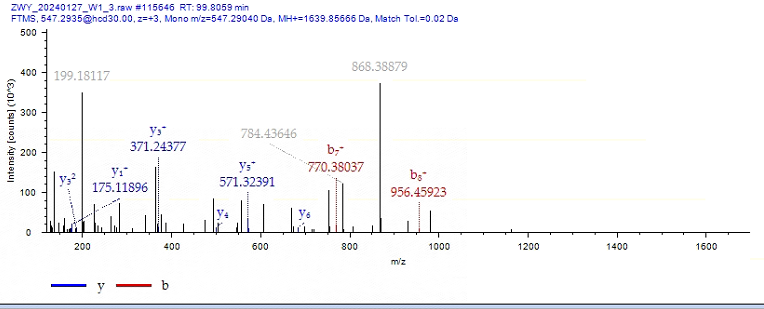


| Dataset | Accession | Peptide | Spectra number |
| --- | --- | --- | --- |
| sh-PTPRJ | P46199 [369-378] | [R].GLVTTAIIQR.[G] | 1 |


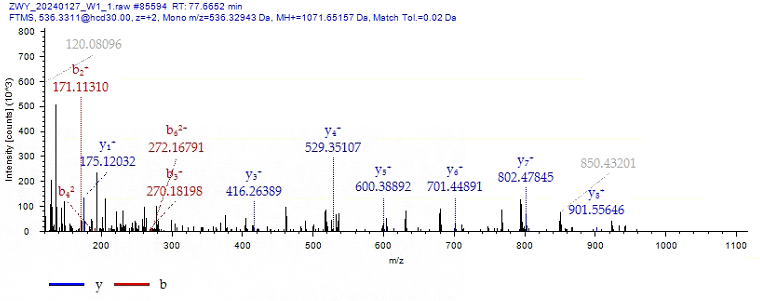


| Dataset | Accession | Peptide | Spectra number |
| --- | --- | --- | --- |
| sh-PTPRJ | O75113 [17-24] | [K].AELLEQSR.[G] | 1 |


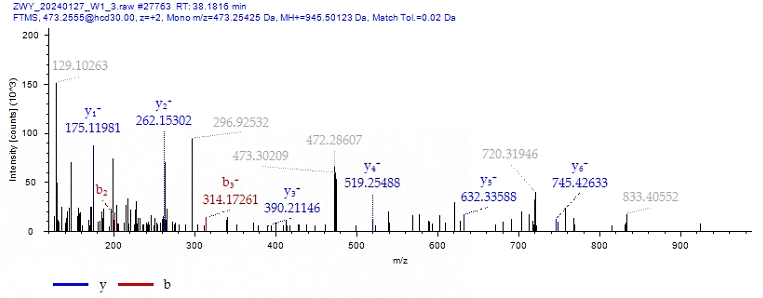


| Dataset | Accession | Peptide | Spectra number |
| --- | --- | --- | --- |
| sh-PTPRJ | P56181 [57-72] | [K].KPAPVPAEPFDNTTYK.[N] | 1 |


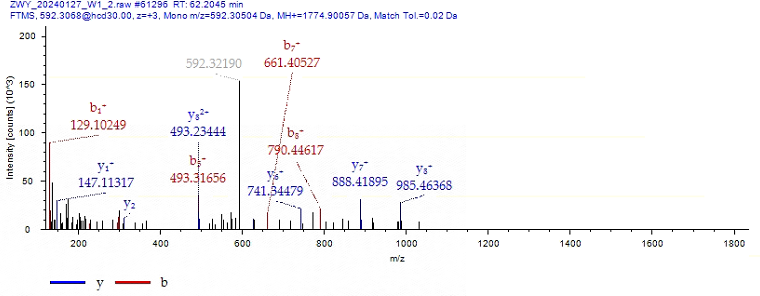


| Dataset | Accession | Peptide | Spectra number |
| --- | --- | --- | --- |
| sh-PTPRJ | P10589 [292-300] | [R].VVAFMDHIR.[I] | 1 |


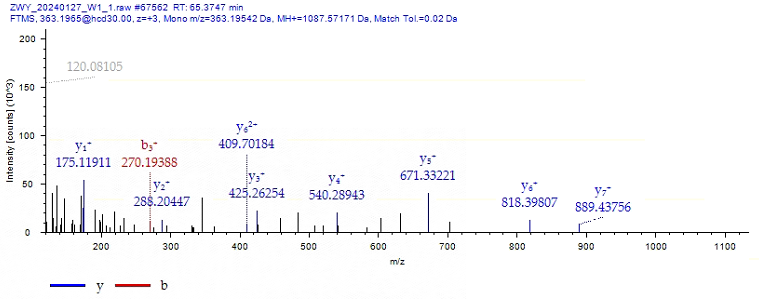


| Dataset | Accession | Peptide | Spectra number |
| --- | --- | --- | --- |
| sh-PTPRJ | P0C646 [129-136] | [K].IGIAAVVR.[S] | 1 |


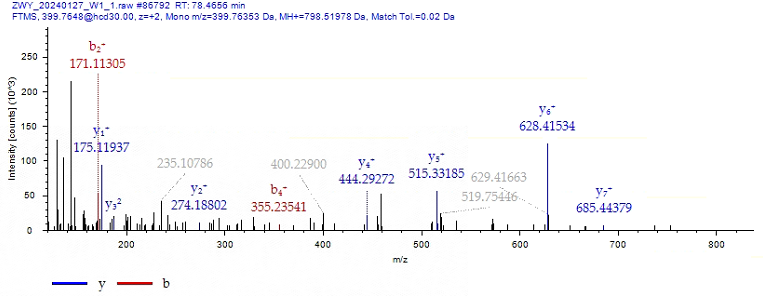


| Dataset | Accession | Peptide | Spectra number |
| --- | --- | --- | --- |
| sh-PTPRJ | O94913 [53-64] | [K].EIVSLIEAQTAK.[A] | 1 |


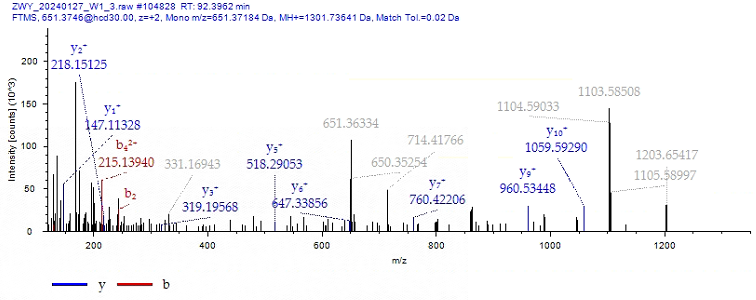


| Dataset | Accession | Peptide | Spectra number |
| --- | --- | --- | --- |
| sh-PTPRJ | Q8WUB8 [207-216] | [K].AAEFNSNLNR.[E] | 1 |


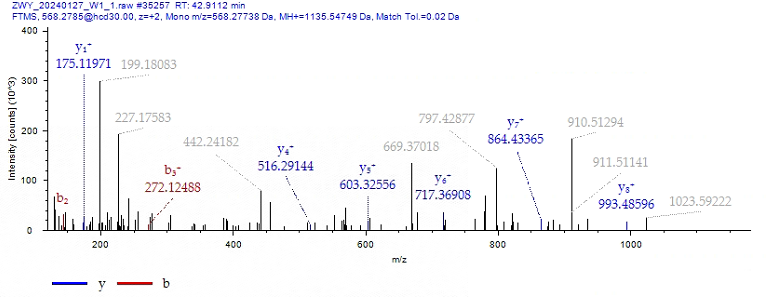


| Dataset | Accession | Peptide | Spectra number |
| --- | --- | --- | --- |
| sh-PTPRJ | Q08623 [72-81] | [K].EELVEESQTK.[L] | 1 |


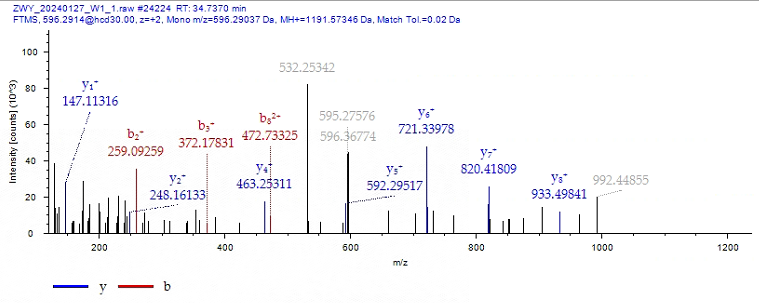


| Dataset | Accession | Peptide | Spectra number |
| --- | --- | --- | --- |
| sh-PTPRJ | Q9NXS2 [70-80] | [R].VPLIGSLPEAR.[L] | 1 |


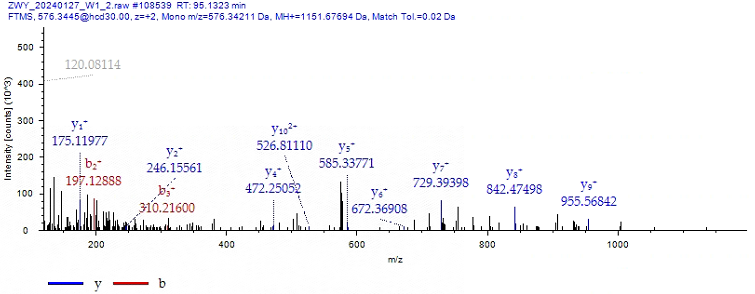


| Dataset | Accession | Peptide | Spectra number |
| --- | --- | --- | --- |
| sh-PTPRJ | O60671 [156-164] | [K].IILQSEGLR.[E] | 1 |


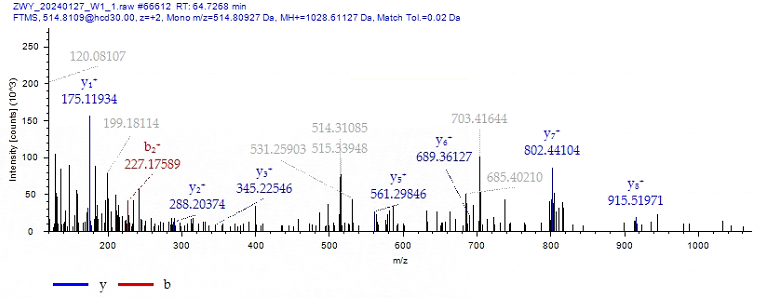


| Dataset | Accession | Peptide | Spectra number |
| --- | --- | --- | --- |
| sh-PTPRJ | Q8WWW0 [335-345] | [K].LSIADRPLYLR.[L] | 1 |


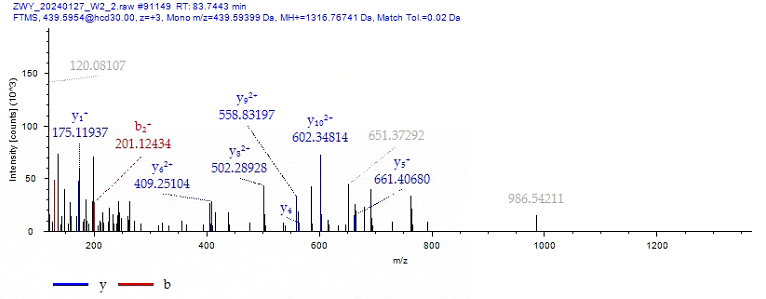


| Dataset | Accession | Peptide | Spectra number |
| --- | --- | --- | --- |
| sh-PTPRJ | Q96HR9 [16-28] | [R].NLVTEVLGALEAK.[T] | 1 |


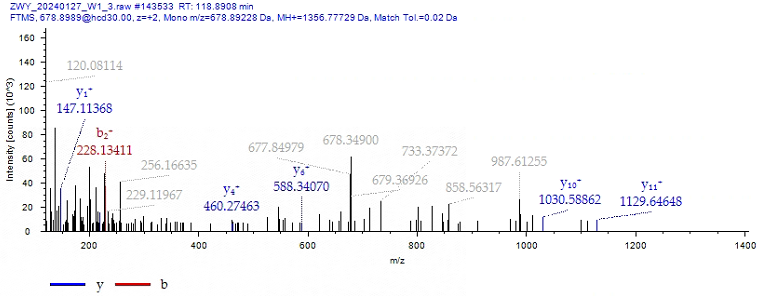


| Dataset | Accession | Peptide | Spectra number |
| --- | --- | --- | --- |
| sh-PTPRJ | O15541 [279-291] | [R].HYFCESCALQHFR.[T] | 1 |


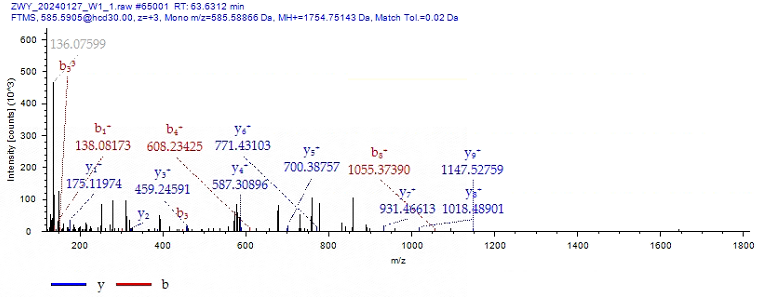


| Dataset | Accession | Peptide | Spectra number |
| --- | --- | --- | --- |
| sh-PTPRJ | Q9HB40 [233-243] | [K].VAEQVLNAVNK.[G] | 1 |


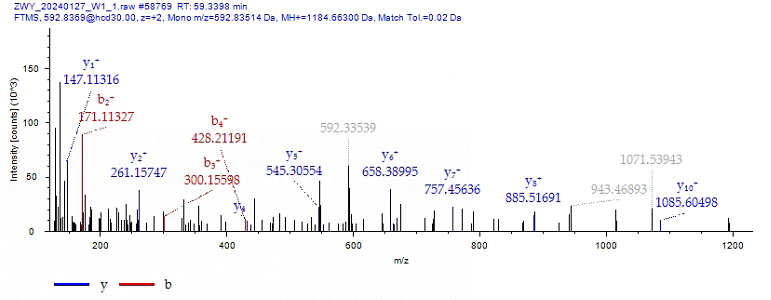


| Dataset | Accession | Peptide | Spectra number |
| --- | --- | --- | --- |
| sh-PTPRJ | O14521 [61-70] | [K].AASLHWTSER.[V] | 1 |


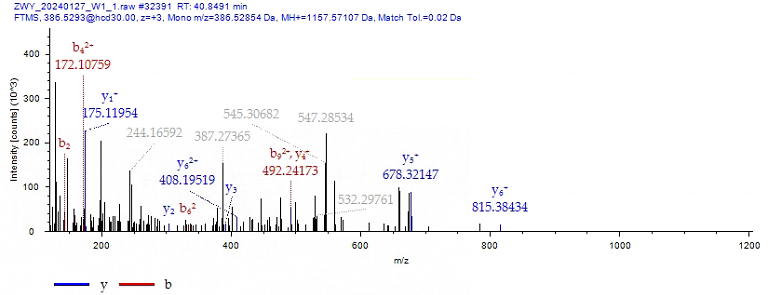


| Dataset | Accession | Peptide | Spectra number |
| --- | --- | --- | --- |
| sh-PTPRJ | Q8TCC7 [292-298] | [R].LSLEELK.[L] | 1 |


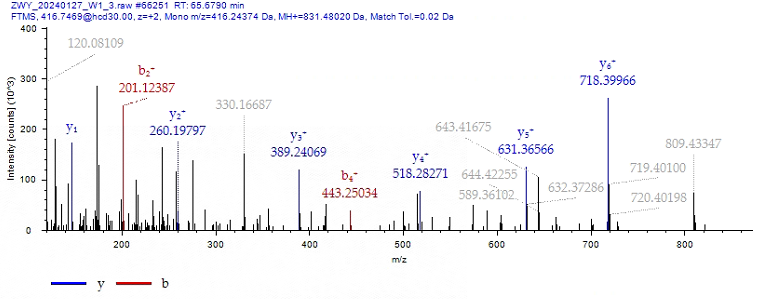


| Dataset | Accession | Peptide | Spectra number |
| --- | --- | --- | --- |
| sh-PTPRJ | P58511 [5-19] | [K].VLEHVPLLLYILAAK.[T] | 1 |


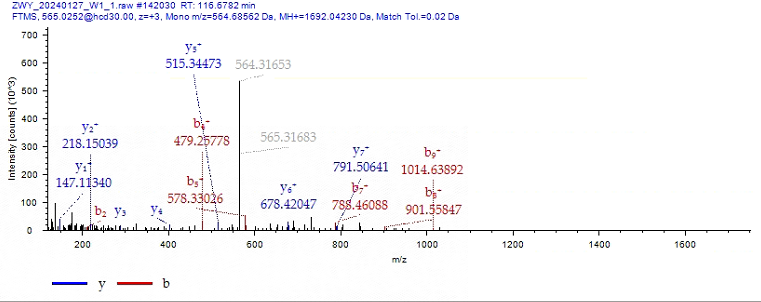


| Dataset | Accession | Peptide | Spectra number |
| --- | --- | --- | --- |
| sh-PTPRJ | O60504 [520-532] | [R].LCDDGPQLPTSPR.[L] | 1 |


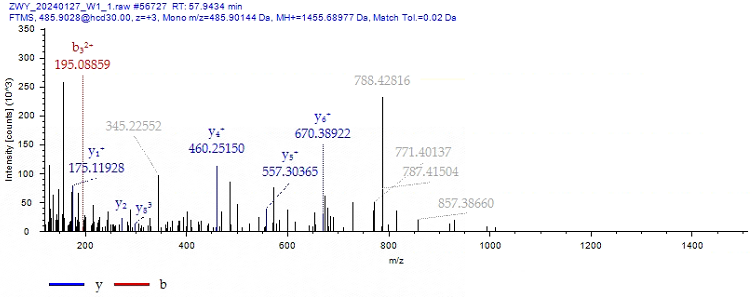


| Dataset | Accession | Peptide | Spectra number |
| --- | --- | --- | --- |
| sh-PTPRJ | P61956 [12-21] | [K].TENNDHINLK.[V] | 1 |


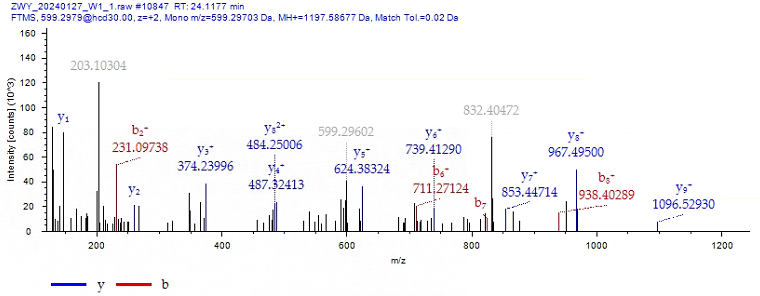


| Dataset | Accession | Peptide | Spectra number |
| --- | --- | --- | --- |
| sh-PTPRJ | Q5T011 [1177-1184] | [K].DLGGTGIK.[A] | 1 |


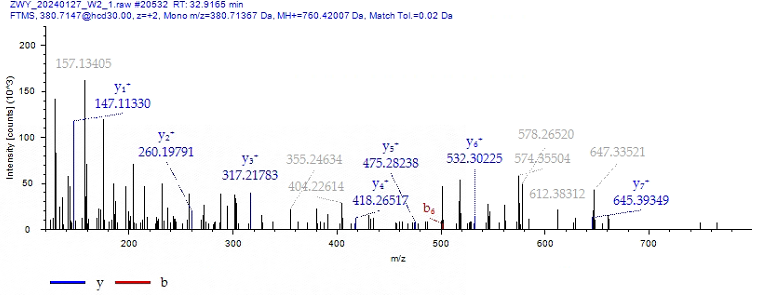


| Dataset | Accession | Peptide | Spectra number |
| --- | --- | --- | --- |
| sh-PTPRJ | Q9Y2S6 [51-59] | [K].GPLATGGIK.[K] | 1 |


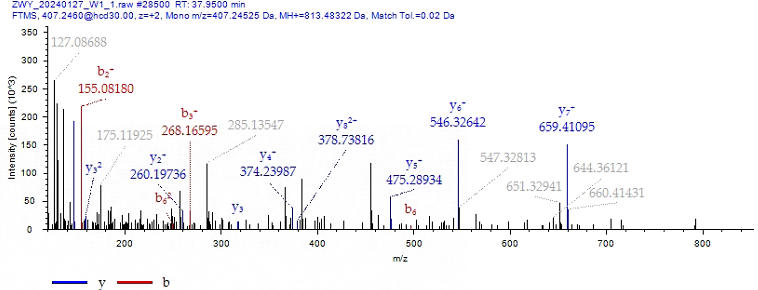


| Dataset | Accession | Peptide | Spectra number |
| --- | --- | --- | --- |
| sh-PTPRJ | Q9NPD8 [70-84] | [R].FLTPIYHPNIDSAGR.[I] | 1 |


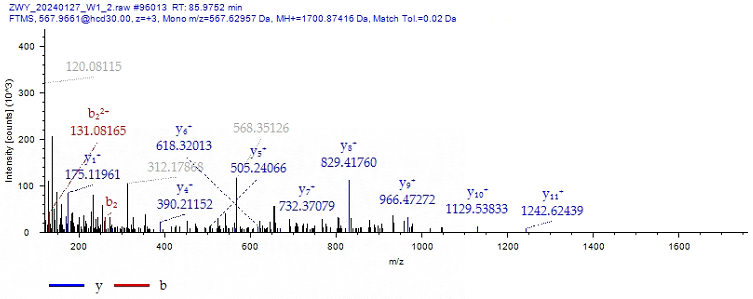


| Dataset | Accession | Peptide | Spectra number |
| --- | --- | --- | --- |
| sh-PTPRJ | O94763 [394-406] | [R].AFVDVVNGEYVPR.[K] | 1 |


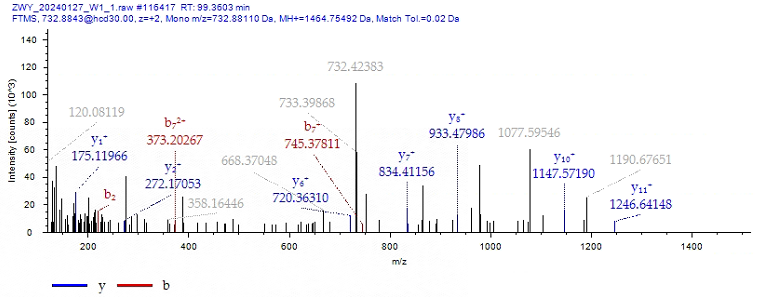


| Dataset | Accession | Peptide | Spectra number |
| --- | --- | --- | --- |
| sh-PTPRJ | Q9Y2C2 [74-79] | [R].FLLDLR.[Q] | 1 |


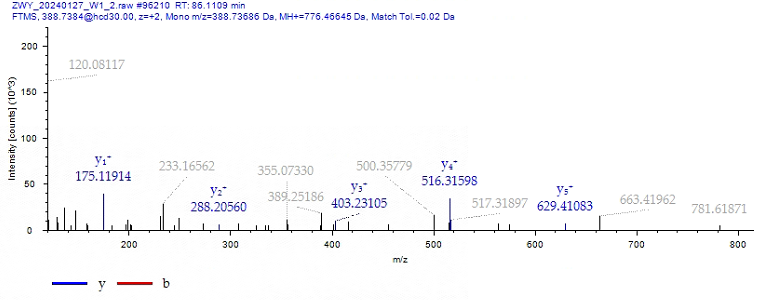


| Dataset | Accession | Peptide | Spectra number |
| --- | --- | --- | --- |
| sh-PTPRJ | P19320 [436-442] | [R].LEIELLK.[G] | 1 |


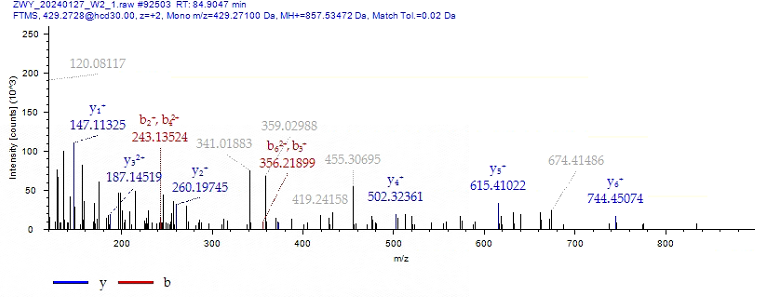


| Dataset | Accession | Peptide | Spectra number |
| --- | --- | --- | --- |
| sh-PTPRJ | Q6P4I2 [139-154] | [R].VAVFSTLAPGVLHGAR.[L] | 1 |


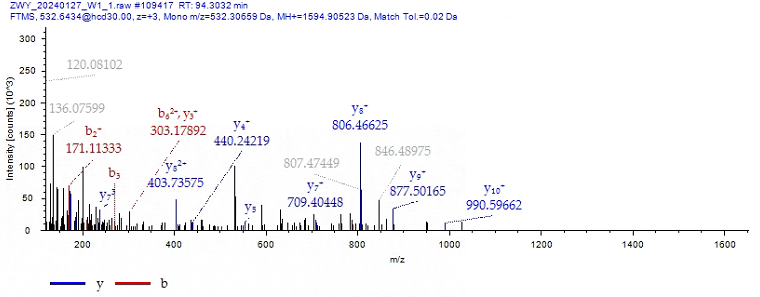


| Dataset | Accession | Peptide | Spectra number |
| --- | --- | --- | --- |
| sh-PTPRJ | Q14966 [118-130] | [K].QSSVTQVTEQSPK.[V] | 1 |


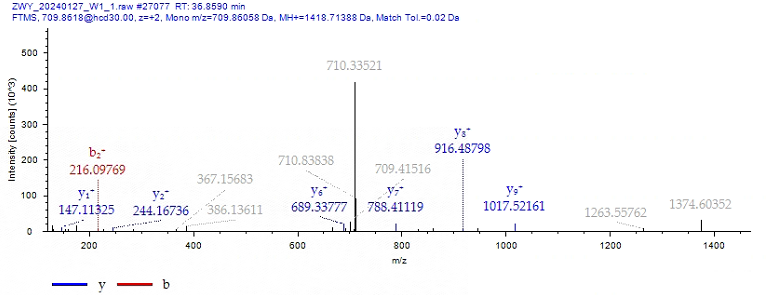

Supplement: Supplemental Data [file mmc3.docx]
